# CD4+ effector T cells accelerate Alzheimer’s disease in mice

**DOI:** 10.1186/s12974-021-02308-7

**Published:** 2021-11-19

**Authors:** Jatin Machhi, Pravin Yeapuri, Yaman Lu, Emma Foster, Rupesh Chikhale, Jonathan Herskovitz, Krista L. Namminga, Katherine E. Olson, Mai Mohamed Abdelmoaty, Ju Gao, Rolen M. Quadros, Tomomi Kiyota, Liang Jingjing, Bhavesh D. Kevadiya, Xinglong Wang, Yutong Liu, Larisa Y. Poluektova, Channabasavaiah B. Gurumurthy, R. Lee Mosley, Howard E. Gendelman

**Affiliations:** 1 Department of Pharmacology and Experimental Neuroscience, University of Nebraska Medical Center, Omaha, NE 68198 USA; 2 Department of Biological Sciences, Northern Kentucky University, Highland Heights, KY 41099 USA; 3 University College London School of Pharmacy, Bloomsbury, London, WC1E 6DE UK; 4 Department of Pathology and Microbiology, University of Nebraska Medical Center, Omaha, NE 68198 USA; 5 Department of Pharmaceutical Sciences, University of Nebraska Medical Center, Omaha, NE 68198 USA; 6 Therapeutic Chemistry Department, National Research Centre, Giza, Egypt; 7 Mouse Genome Engineering Core Facility, University of Nebraska Medical Center, Omaha, NE USA; 8 Department of Safety Assessment, Genentech Inc., South San Francisco, CA 94080 USA; 9 Department of Radiology, University of Nebraska Medical Center, Omaha, NE 68198 USA

**Keywords:** Alzheimer’s disease (AD), Amyloid beta (Aβ), T cell, Effector T cell (Teff), Regulatory T cell (Treg), APP/PS1 transgenic mice

## Abstract

**Background:**

Alzheimer’s disease (AD) is a progressive neurodegenerative disorder characterized by pathological deposition of misfolded self-protein amyloid beta (Aβ) which in kind facilitates tau aggregation and neurodegeneration. Neuroinflammation is accepted as a key disease driver caused by innate microglia activation. Recently, adaptive immune alterations have been uncovered that begin early and persist throughout the disease. How these occur and whether they can be harnessed to halt disease progress is unclear. We propose that self-antigens would induct autoreactive effector T cells (Teffs) that drive pro-inflammatory and neurodestructive immunity leading to cognitive impairments. Here, we investigated the role of effector immunity and how it could affect cellular-level disease pathobiology in an AD animal model.

**Methods:**

In this report, we developed and characterized cloned lines of amyloid beta (Aβ) reactive type 1 T helper (Th1) and type 17 Th (Th17) cells to study their role in AD pathogenesis. The cellular phenotype and antigen-specificity of Aβ-specific Th1 and Th17 clones were confirmed using flow cytometry, immunoblot staining and Aβ T cell epitope loaded haplotype-matched major histocompatibility complex II IA^b^ (MHCII-IA^b^–KLVFFAEDVGSNKGA) tetramer binding. Aβ-Th1 and Aβ-Th17 clones were adoptively transferred into APP/PS1 double-transgenic mice expressing chimeric mouse/human amyloid precursor protein and mutant human presenilin 1, and the mice were assessed for memory impairments. Finally, blood, spleen, lymph nodes and brain were harvested for immunological, biochemical, and histological analyses.

**Results:**

The propagated Aβ-Th1 and Aβ-Th17 clones were confirmed stable and long-lived. Treatment of APP/PS1 mice with Aβ reactive Teffs accelerated memory impairment and systemic inflammation, increased amyloid burden, elevated microglia activation, and exacerbated neuroinflammation. Both Th1 and Th17 Aβ-reactive Teffs progressed AD pathology by downregulating anti-inflammatory and immunosuppressive regulatory T cells (Tregs) as recorded in the periphery and within the central nervous system.

**Conclusions:**

These results underscore an important pathological role for CD4+ Teffs in AD progression. We posit that aberrant disease-associated effector T cell immune responses can be controlled. One solution is by Aβ reactive Tregs.

**Graphical Abstract:**

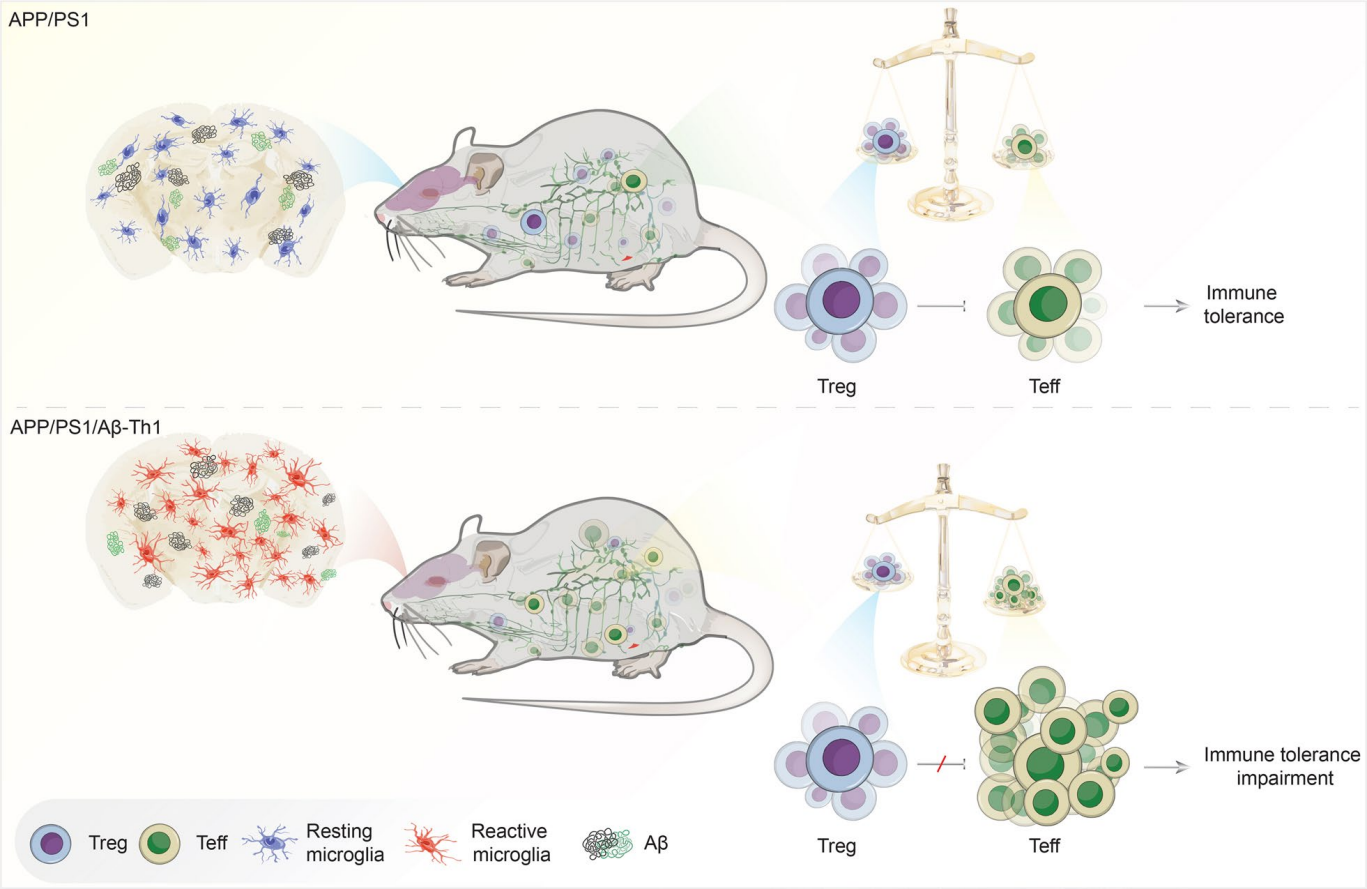

**Supplementary Information:**

The online version contains supplementary material available at 10.1186/s12974-021-02308-7.

## Background

Alzheimer’s disease (AD) is the most common form of age-associated dementia and has become the sixth leading cause of death in the United States [1, 2]. AD is characterized by the presence of misfolded self-proteins. Those self-proteins include amyloid beta (Aβ) and hyperphosphorylated tau present within the brain and the systemic circulation [3, 4]. Aβ and tau aggregate and deposit among brain tissues as senile or amyloid plaques and neurofibrillary tangles. In addition, neuroinflammation persists and has been identified as a key disease-driving factor [2, 5]. Both innate and adaptive immunity play disease-propagating roles [2, 6, 7]. Central nervous system (CNS) innate microglial cells and astrocytes are activated by the disease proteins and contribute to neuronal injuries by producing inflammatory and neurotoxic factors. These increase aberrant amyloid precursor protein (APP) cleavage, Aβ production, and tau phosphorylation [8, 9]. The role of adaptive immune T cells in AD pathogenesis is under-reported and less well understood. We posit that understanding disease-linked adaptive immunity will permit the harnessing of peripheral immune T cell responses to facilitate AD therapeutic management.

The peripheral adaptive immune system, comprised of cytokine secreting T cells, monocytes, and B lymphocytes, is activated by modified self-peptide fragments, such as Aβ and tau. These are engulfed and presented by antigen presenting cells (APCs) [2, 10]. T cell subsets contribute either effector or regulatory functions that are maintained during homeostatic conditions to maintain immunological tolerance. However, in pathological states, effector T cell (Teff) subsets react against misfolded and disease-specific self-proteins that can break immune tolerance and expand self-reactive T cells [2, 11, 12]. Clonal expansion of CD8+ Teffs in both the periphery and CNS has been shown in AD patients [13]. Similarly, elevated CD4+ Teffs are reported in AD patients with mild cognitive impairment [14–16]. Additionally, CD4+ Teff subsets reactive against AD-associated pathological proteins that included Aβ-reactive type 1 T helper (Th1), type 2 Th (Th2), and type 17 Th (Th17) cells were studied in mixed microglia–astroglia cultures. These studies demonstrated that glial pro-inflammatory responses are driven by Th1 and Th17 and regulated by Th2 cells [17]. Among Aβ responsive Th1, Th2, and Th17 cells, only interferon gamma (IFNγ)-secreting Th1 cells accelerate disease in double transgenic mice expressing chimeric mouse/human amyloid precursor protein and mutant human presenilin 1 (APP/PS1). Disease can be corrected by passive transfer of anti-IFNγ antibodies [18]. In contrast, protective effects of intracerebroventricularly injected Aβ-specific Th1 cells in 5XFAD (C57BL6) mice facilitate enhancement of microglial major histocompatibility complex class II (MHCII) and increased phagocytic activities [19, 20]. Overall, the role played by CD4+ Teffs in AD pathogenesis remains incompletely understood. In this report, we investigated the role of Teffs in AD-associated brain pathologies by developing Aβ-specific CD4+ Th1 and Th17 Teff clonal cell lines, used to study the role of Aβ-specific Teffs in AD progression. The unique cytokine signature, transcription factor expression, and MHCII-IA^b^-Aβ tetramer staining affirmed a distinctive cellular phenotype and antigen specificity. Aβ-reactive Th1 and Th17 Teffs, adoptively transferred into APP/PS1 mice demonstrated accelerated behavioral and pathological disease, thus supporting a role as disease perpetrators. This suggests an evolving role for CD4+ Teffs in AD progression with opportunities for therapeutic interventions by immune modulation.

## Results

### Aβ-Th1 and Aβ-Th17 cell phenotypes are stable

**Fig. 1:**
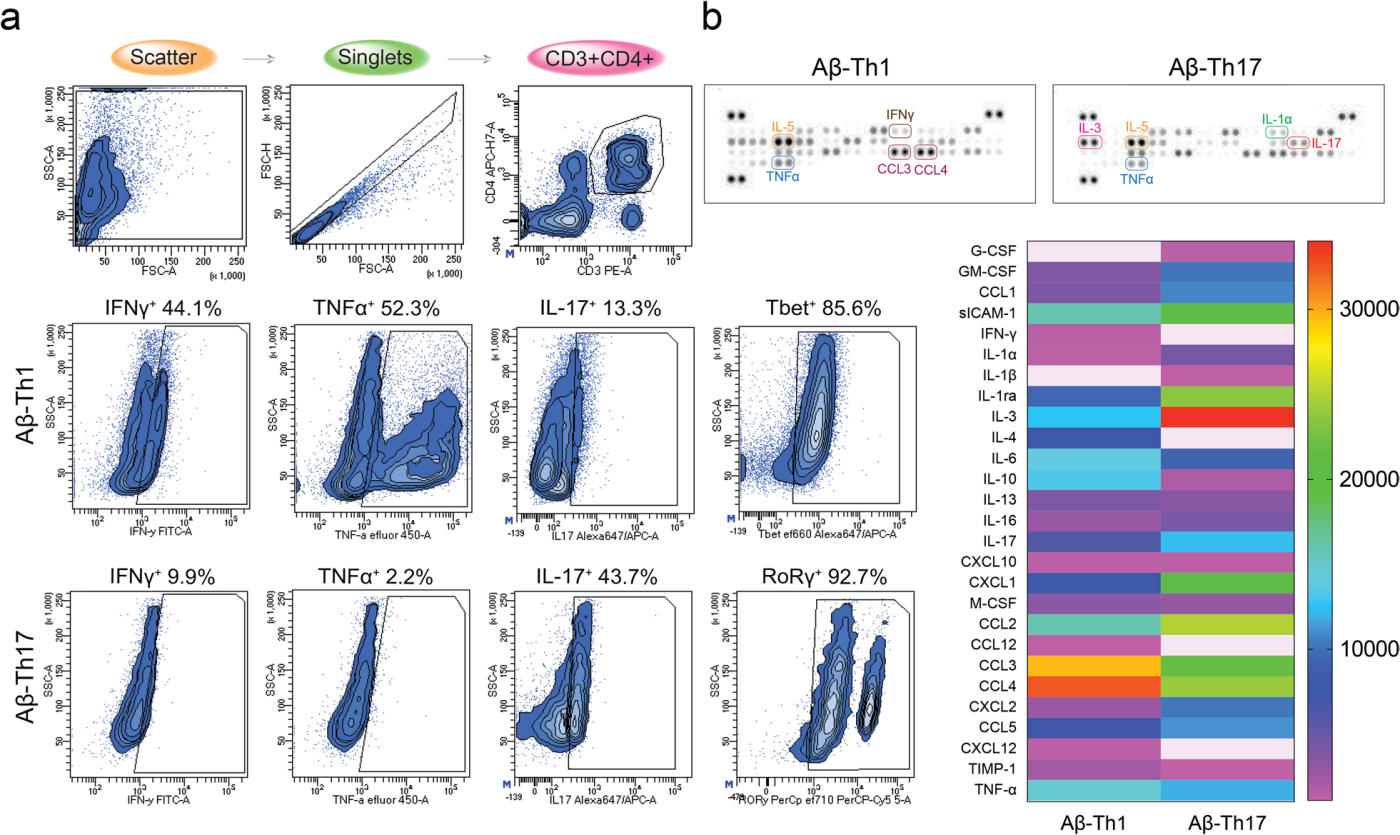
Cellular phenotype of the Aβ-Th1 and Aβ-Th17 cells. **a** Flow cytometric analysis of intracellular cytokine and transcription factor expressed by Aβ-Th1 and Aβ-Th17 cells that were maintained as clones for greater than 6 months. T cells were stimulated for 12 h with PMA and ionomycin in the presence of brefeldin A. **b** Representative immunoblot and quantification for 42 different cytokines and chemokines extracellularly secreted from Aβ-Th1 and Aβ-Th17 cells after stimulation with PMA and ionomycin

**Fig. 2:**
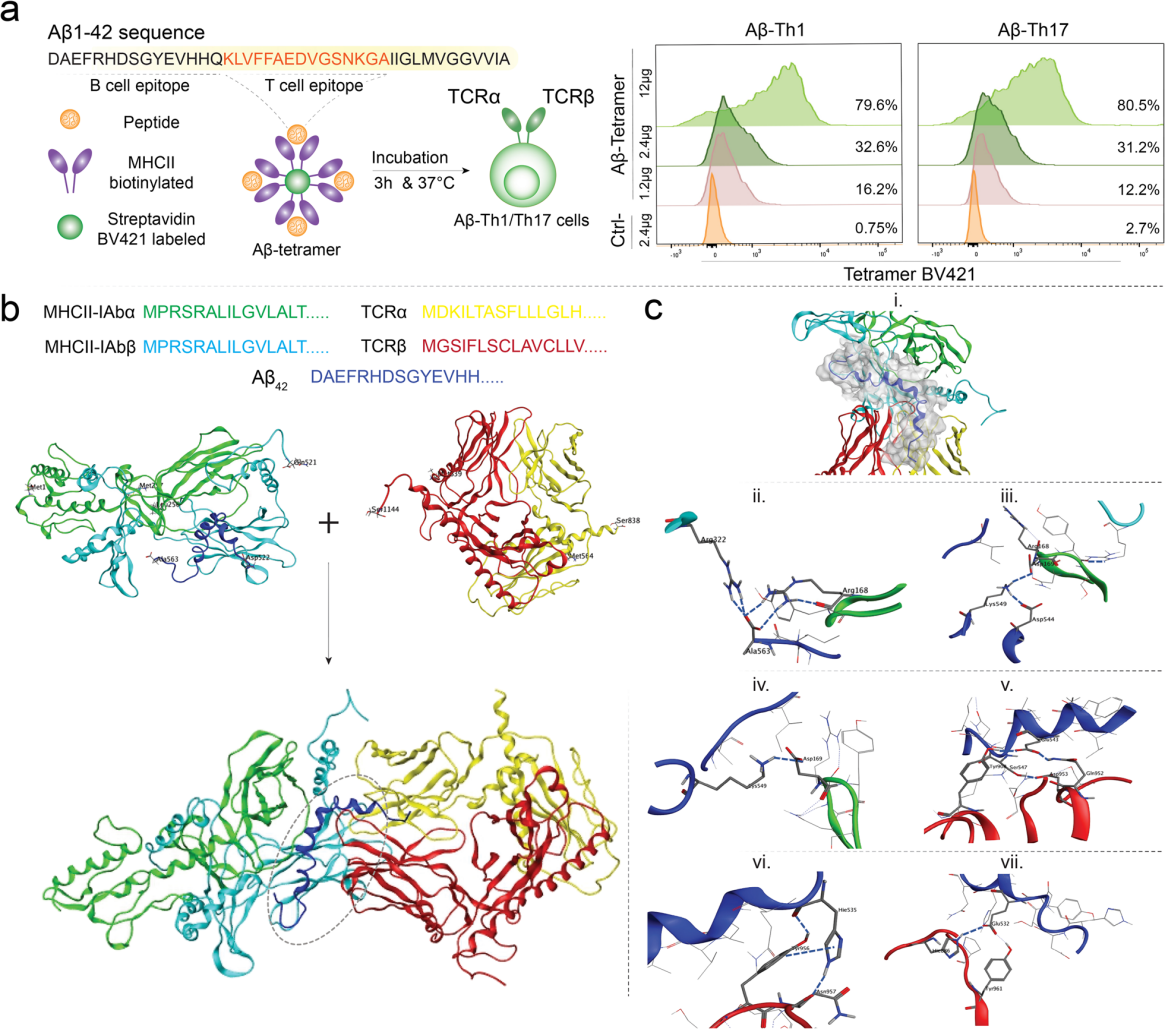
Antigen-specificity of the Aβ-Th1 and Aβ-Th17 cells **a** MHCII-IA^b^–KLVFFAEDVGSNKGA (Aβ T cell epitope) tetramer binding with Aβ-Th1 and Aβ-Th17 cells after incubation. Control tetramer MHCII-IA^b^–PVSKMRMATPLLMQA was used for precise gating of Aβ T cell epitope recognizing CD4+ T cell population. **b** From Aβ-Th1 cells, antigen-recognizing variable regions of T cell receptor (TCR) alpha (α) and beta (β) chains were identified using molecular cloning. Molecular modeling of full-length TCRα/β complex with Aβ_1–42_–MHCII-IA^b^ (pMHC) complex; MHCII-IA^b^α chain (green), MHCII-IA^b^β chain (cyan) and peptide (blue); TCRα chain (yellow) and TCRβ chain (red). The interface of MHCII, peptide and TCR binding is shown by encircled region. **c** (i) peptide surface at the interface of MHC and TCR. Peptide–MHC interactions; (ii) peptide Ala563 and Arg322 interaction with MHCα Arg168 and MHCβ Arg322, respectively; (iii) peptide Lys549 interaction with MHCα Arg168(O); (iv) peptide Lys549 interaction with MHCα Asp169(OD2); TCR–pMHC interactions- (v) pMHC Glu543 and Ser547 interaction with Tyr908, Asp953 and Gln952. (vi) pMHC His535 (NE2) interaction with Asn(O), and His535 (O) interaction with Tyr956 (OH), π–π interactions between Tyr956 and His535; (vii) pMHC interaction with TCRβ, Glu532(OE2)–Tyr961(OH) and Glu532 (OE1)–His886(NE2)

Previous works developed antigen-specific T cells from Aβ_1–42_ immunization of non-transgenic (non-Tg) mice. However, those cells were polarized for a short period prior to adoptive transfer with uncertain phenotypes [18, 19]. Here, we developed stable Aβ-specific Teff clones that were maintained in culture for more than 6 months and retained antigen specificity and phenotype. Flow cytometric analysis for intracellular cytokines revealed that activated Aβ-Th1 cells expressed pro-inflammatory cytokines, interferon gamma (IFNγ) (44.1%) and tumor necrosis factor alpha (TNFα) (52.3%), and expressed nuclear transcription factor T-box expressed in T cells (Tbet) (85.6%). Aβ-Th17 cells preferentially expressed interleukin 17 (IL17) (43.7%) and transcription factor RAR-related orphan receptor gamma (RORγ) (92.7%) (Fig. 1a). To further confirm the cellular phenotype of Aβ-Teffs, extracellular cytokine release was assessed by the immunoblot staining, which showed that Aβ-Th1 cells selectively secreted IFNγ, while Aβ-Th17 cells secreted higher levels of IL17 (Fig. 1b). Both Aβ-Th1 and Aβ-Th17 cells secreted TNFα after activation, although higher expression was observed in Aβ-Th1 cells. Aβ-Th1 and Aβ-Th17 cells also upregulated chemokines CCL3 (MIP-1α) and CCL4 (MIP-1β), but expression was 35% higher in Aβ-Th1 cells compared to Aβ-Th17 cells. CCL3 and CCL4 have been implicated in different inflammatory conditions and are produced in substantial quantities by the Th1 type lymphocytes [21], further supporting the cellular phenotype of Aβ-Teffs. The unique T cell receptor (TCR) recognizes cognate antigen when presented by the major histocompatibility complex type I or type II (MCH-I or -II) molecules of antigen presenting cells (APCs) [2, 10]. To determine the antigen specificity of Teff clones, Aβ-Th1 and Aβ-Th17 cells were incubated with fluorescently labeled MHCII-IA^b^–KLVFFAEDVGSNKGA tetramer. The 3-month Aβ-Th1 and Aβ-Th17 clones showed binding with MHC–peptide tetramer in a dose-dependent manner, confirming their Aβ specificity (Additional file 1: Fig. S1b). Prolonged maintenance of stable Teff clones (6 months) maintained their cognate antigen recognition seen through dose-dependent Aβ–Teff–MHC–peptide binding in Fig. 2a comparable to the 3-month clones (Additional file 1: Fig. S1b).

To provide greater insight in parent Th1 clone interaction with Aβ peptide bound to MHCII-IA^b^, TCRα and TCRβ chain sequences were identified, and molecular modeling studies were performed with peptide–MHCII (pMHC) complex (Fig. 2b). Molecular modeling of full-length TCRα/β complex with Aβ_1–42_–MHCII–IA^b^ (pMHCII) complex was performed to determine exact amino acid interactions between two complexes [22]. The model was constructed with structural assessment, validation for various features, bond orientation, and overall quality of model performed. pMHC complex consisted of MHCII-IA^b^α (green chain), MHCII-IA^b^β (cyan chain), and Aβ peptide (blue chain) (Fig. 2b). TCR complex consisted of TCRα (yellow chain) and TCRβ (red chain). The Ramachandran plot or the φ/ψ plot predicts the conformational stability of the models, the TCR–pMHC complex had a RC score of 98.35% for favored region and 0.44% for outliers suggested its good quality (Additional file 1: Fig. S2a(i,ii)). MolProbity score and the QMEAN score reflects overall quality of the model, herein they were 0.66 and −3.60, respectively. The TCR–pMHC complex was subjected to molecular dynamics (MD) simulations and the final structure from the 100 ns MD trajectory was analyzed for its root-mean-square deviation (RMSD) and root-mean-square fluctuations (RMSF) for individual amino acid residues [23]. The RMSD during the MD simulation for whole complex gradually rose by 5 Å from 2 to 7 Å during the initial 40 ns and then converged to stabilize between 1.5 Å (5.5 to 7 Å) for rest of the simulation (Additional file 1: Fig. S2a(iii)). The RMSF during the simulation was observed between 1 to 14 Å with most of the residues having RMSF between 1 to 3 Å (Additional file 1: Fig. S2a(iv)). The individual components of the complex were studied and the RMSF for each component was analyzed. MHCα and MHCβ are green and cyan colored ribbons, respectively (Fig. 2b(i)), MHCα had RMSF of 3 Å for N-terminal Met1 and about 2 Å for C-terminal Glu73 (Additional file 1: Fig. S3b(ii)), the residues belonging to the loop regions (residues 40–45) had very high RMSF of about 8.5 Å. The MHCβ had RMSF of 2.25 Å for N-terminal Met257 and about 4.5 Å for C-terminal Gln521 (Additional file 1: Fig. S2b(iii)). Aβ peptide N-terminal residue Asp522 and C-terminal residue Ala563 showed interactions with MHC and TCR molecules (Fig. 2b(i)). Stabilized fluctuations and formation of multiple interactions between peptide–MHC and pMHC–TCR are shown under the surface diagram (Fig. 2c(i)). MHCα and MHCβ showed multiple interactions with Aβ peptide; Ala563 of peptide interacted with Arg168 of MHCα chain and Arg322 of MHCβ chain to form donor–receptor pair leading to hydrogen bonds formation (Fig. 2c(ii)). Aβ residue Lys549 formed hydrogen bond interaction with backbone of MHCα chain Arg168(O) (Fig. 2c(iii)) and with MHCα chain Asp169(OD2) (Fig. 2c(iv)). TCR chains were analyzed for their RMSF; TCRα chain showed higher fluctuations of around 6 Å owing to terminal residues, but TCRβ chain had lower RMSF between 1 and 3 Å for most of the residues with the terminal residues showing about 10 Å, which was expected for the terminal helixes. The Fab (fragment antigen binding) region showed lower RMSF due to its complexation with pMHC molecule (Additional file 1: Fig. S2b(i–iii)). pMHC molecule predominantly interacted with TCRβ chain and formed multiple interactions; pMHC sidechain residues Glu543 and Ser547 formed hydrogen bonds with Tyr908, Asp953 and Gln952 of TCRβ (Fig. 2c(v)). pMHC residue His535 (NE2) formed hydrogen bond with Asn(O). His535 (O) interacted with Tyr956 (OH) to form intermolecular hydrogen bonding and due to stacking of the aromatic rings Tyr956 and His535 also showed π–π interactions (Fig. 2c(vi)); pMHC peptide residue Glu532 also interacted with TCRβ, Glu532(OE2)–Tyr961(OH) and Glu532 (OE1)–His886(NE2) (Fig. 2c(vii)). Higher fluctuations in both TCRα and TCRβ chains were observed for the Fc (fragment crystallizable) regions, which were attributed to the helices that form C-terminals of TCRα and TCRβ chains (Additional file 1: Fig. S2c(i–v)). The overall TCR–pMHC model suggests interaction of Aβ peptide in the region between residues Glu532 to Ala563 with MHC molecule and TCR Fab region, with rest of the peptide having very less to no interactions during the MD simulation of the complex.

### Adoptive transfer of Aβ-Teffs accelerates memory impairment in mice

**Fig. 3:**
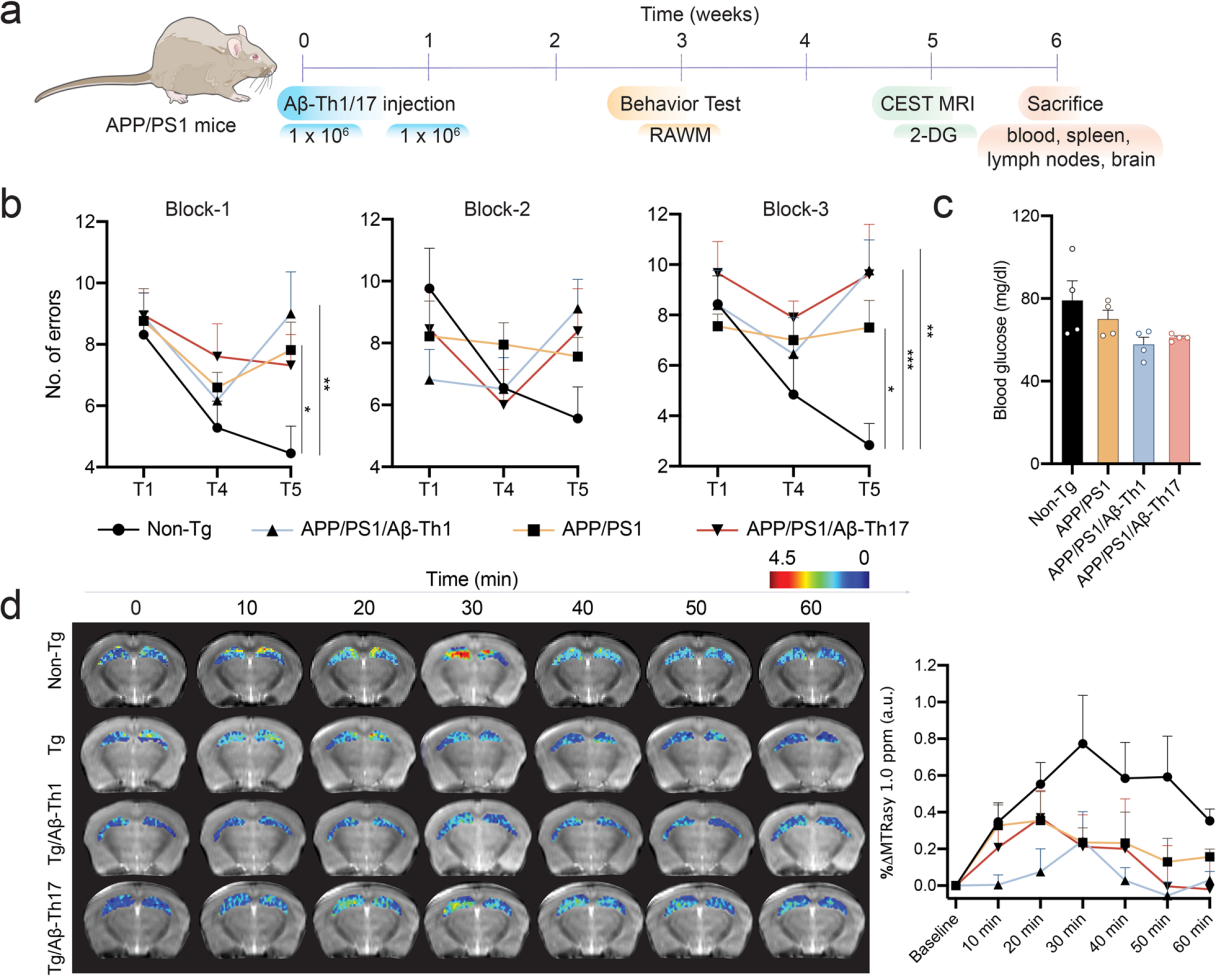
Aβ-Th1 and Aβ-Th17 cells affect memory function in APP/PS1 mice. **a** Schematic presentation of the in vivo experimental procedure performed in 4- to 5-month-old female APP/PS1 and age-matched non-Tg mice. *n* = 6 mice per group were used. **b** Radial arm water maze (RAWM) test was performed with experimental mice 3 weeks after the first of two adoptive transfers with 1 × 10^6^ Aβ-Th1 or Aβ-Th17 cells intravenously. Errors of 9-day trials were divided into three blocks and averaged for the statistical analysis. Two-way ANOVA was used to determine significant differences between experimental groups. *p < 0.05, **p < 0.01. **c** Fasting blood glucose concentrations were measured prior to the glucoCEST MRI. **d** 2-deoxy-glucose (2DG) CEST MRI was performed 5 weeks after the Aβ-Th1 and Aβ-Th17 cells adoptive transfer. Representative MRI images with hippocampal glucose signal are shown for different experimental mice which include baseline MRI scan followed by 2DG injection and thereafter MRI scans every 10 min up to 1 h. *n* = 4 mice per group were analyzed. Glucose intensity in the hippocampus was calculated as %ΔMTR at different time points compared to the baseline. Area under the curve (AUC) for each mouse was calculated and averaged for statistical significance using one-way ANOVA followed by Newman–Keuls post hoc test. Same line colors and symbols are used to show different groups in **b** and **d**

To test the behavioral effects of Aβ-Teffs, 2 × 10^6^ Aβ-Th1 or Aβ-Th17 cells were adoptively transferred to APP/PS1 mice in 2 weekly doses and recipients were evaluated for spatial learning and memory in the radial arm water maze (RAWM) test 2 weeks after second adoptive transfer (Fig. 3a). APP/PS1 mice showed signs of memory impairment evidenced by significant increases in the number of errors in late retention trial T5 (*p* < 0.05, block-1 and block-3) compared to non-Tg mice (Fig. 3b) and were consistent with our previous reports [6]. Higher number of memory errors were observed in APP/PS1 recipients that received either Aβ-Th1 or Aβ-Th17 cells compared to non-Tg mice (Aβ-Th1 *p* < 0.01 in block-1 and *p* < 0.001 in block-3 while Aβ-Th17 *p* < 0.01 in block-3). Additionally, APP/PS1 mice that received Aβ-Teffs exhibited 30% greater number of experimental errors compared to untreated APP/PS1 mice, but were not significantly different. Overall, these results support the notion that Aβ-Th1 and Aβ-Th17 Teffs increase memory impairment development speed in APP/PS1 mice and is linked to a pro-inflammatory phenotype under an amyloid enriched environment.

Alteration in brain glucose uptake and its subsequent metabolism is a biomarker for memory impairment [24, 25], and has been used to confirm the effects of Aβ-Teffs on the memory functions of APP/PS1 mice. ^18^F-radiolabeled fluorodeoxyglucose (^18^F-FDG) positron emission tomography (PET) imaging is commonly used for the diagnosis of dementia stage in the AD patients [26, 27] and in laboratory animal models [28, 29], whereas non-radiolabeled 2-deoxy glucose (2DG) chemical exchange saturation transfer (CEST)-MRI has been shown to assess the glucose uptake in AD animal models with better resolution [30]. 2DG can quickly enter the brain via the same transporters for the D-glucose, where it is metabolized into 2DG-6-phosphate (2DG6P), but minimally metabolized further. Due to low blood–brain barrier (BBB) permeability, 2DG6P is trapped inside the brain that allows assessment of glucose uptake by the CEST-MRI approach as described earlier [31]. Prior to glucoCEST, fasting blood glucose concentration was reduced in APP/PS1 mice (11%) compared to non-Tg mice, whereas concentrations in APP/PS1/Aβ-Th1 and APP/PS1/Aβ-Th17 mice decreased by 27% and 23%, respectively (Fig. 3c). Non-Tg mice showed highest 2DG uptake as glucoCEST signal in the hippocampus, a region that primarily drives the memory functions, and then slowly declined over more than 1 h as shown in the representative scans (Fig. 3d). APP/PS1 mice showed a low hippocampal glucoCEST signal compared to non-Tg mice. The area under curve (AUC) of glucoCEST for APP/PS1 mice was 55% lower than non-Tg mice. The AUC was further reduced in APP/PS1/Aβ-Th1 mice (*p* < 0.01, 87%). APP/PS1/Aβ-Th17 mice showed 67% less AUC compared to non-Tg mice. APP/PS1/Aβ-Th1 and APP/PS1/Aβ-Th17 mice decreased AUC 72% and 26%, respectively, compared to untreated APP/PS1 mice, but did not achieve significance. Thus, Aβ-Teffs decreased glucose uptake in the hippocampus region of APP/PS1 mice paralleled their abilities to accelerate memory impairment.

### Aβ-Teffs promote systemic inflammation

**Fig. 4:**
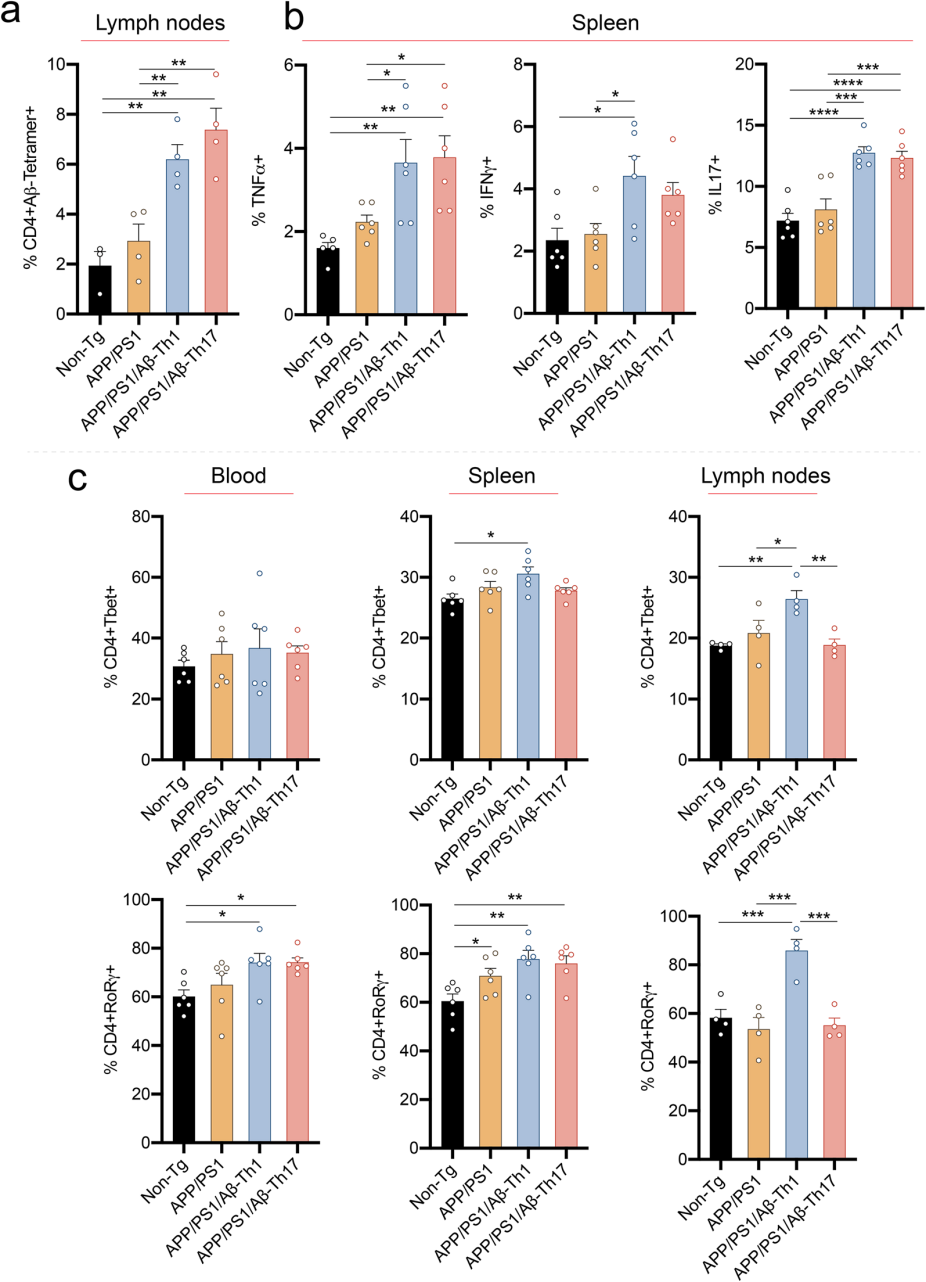
Adoptive transfer Aβ-Teffs affect systemic inflammation in APP/PS1 mice. Experimental mice were killed 6 weeks after Aβ-Th1 and Aβ-Th17 adoptive transfer. **a** Frequency of Aβ reactive CD4+ T cells among lymph node cells after stimulation with Aβ_1–42_ in presence of feeder cells using fluorescently labeled MHCII-IA^b^-peptide tetramer. *n* = 4 mice per group were analyzed. **b** Frequency of intracellular pro-inflammatory cytokines TNFα, IFNγ and IL17 from splenocytes after stimulation with PMA and ionomycin in the presence of brefeldin A. *n* = 6 mice per group were analyzed. **c** Frequency of CD4+Tbet+ and CD4+RORγ+ T cells in blood, spleen and lymph nodes determined by flow cytometric analysis. *n* = 6 mice per group were analyzed. One-way ANOVA followed by Newman–Keuls post hoc test was used to determine statistical significance. *p < 0.05, **p < 0.01, ***p < 0.001, ****p < 0.0001

The onset of systemic inflammatory responses has been demonstrated decades before memory impairment and subsequent neurodegeneration in AD [32]. Moreover, higher levels of serum inflammatory cytokines are observed in individuals who after 20 years demonstrated memory decline and decreased brain volume [33]. Such observations suggest that systemic inflammatory responses play an important early role in AD pathology before amyloid deposition and microglia activation in the brain [2, 14, 15]. To determine the effects of Aβ-Teffs on systemic inflammation, frequencies of different T cell subsets and inflammatory cytokines were measured in blood, spleen and lymph nodes after adoptive transfer. Although, frequencies of CD4+ and CD8+ T cells were unchanged regardless of mouse strain or treatment (Additional file 1: Fig. S3), the subset analysis revealed increased systemic pro-inflammatory markers. It was hypothesized that systemic inflammatory responses predominantly arise from antigen-specific T cells present in the periphery of experimental mice. Therefore, we tested the frequency of Aβ reactive CD4+ T cells by stimulating lymph node cells with cognate antigen (Aβ) in presence of APCs. CD4+ T cells from non-Tg mice showed very low binding with MHCII–peptide tetramer, suggesting the presence of few Aβ-CD4+ T cells during homeostasis (Fig. 4a). In untreated APP/PS1 mice, Aβ-CD4+ T cell frequency was slightly, though insignificantly, higher compared to non-Tg mice (Fig. 4a). However, APP/PS1/Aβ-Th1 and APP/PS1/Aβ-Th17 mice exhibited significantly higher levels of Aβ-MHCII tetramer reactive CD4+ T cells compared to non-Tg and APP/PS1 mice (*p* < 0.01). The results indicate that antigen-recognizing CD4+ T cell populations are sustained after adoptive transfer of Aβ-Teffs and suggested a contributory role in systemic inflammation associated with AD. Indeed, splenocytes isolated from APP/PS1/Aβ-Th1 and APP/PS1/Aβ-Th17 mice and stimulated with phorbol-12-myristate-13-acetate (PMA) and ionomycin secreted significantly higher levels of pro-inflammatory cytokines TNFα (*p* < 0.05) and IL17 (*p* < 0.001) compared to splenocytes isolated from non-Tg and untreated APP/PS1 mice (Fig. 4b). Additionally, APP/PS1/Aβ-Th1 mice secreted significantly higher levels of IFNγ (*p* < 0.05) compared to non-Tg and untreated APP/PS1 mice. Compared to non-Tg controls, no differences in CD4+Tbet+ T cell frequencies from APP/PS1 mice in any lymphoid compartment were detected, while CD4+RORγ+ T cell frequency was significantly increased in spleens of APP/PS1 mice (*p* < 0.05) (Fig. 4c). CD4+Tbet+ T cells were significantly elevated in spleen (*p* < 0.05) and lymph node (*p* < 0.01) of APP/PS1/Aβ-Th1 mice compared to non-Tg counterparts. Similarly, CD4+RORγ+ T cell frequencies were significantly increased in all tested lymphoid compartments of APP/PS1/Aβ-Th1 mice (*p* < 0.01) while in APP/PS1/Aβ-Th17 mice, CD4+RORγ+ T cell frequencies were significantly increased in blood (*p* < 0.05) and spleen (*p* < 0.01) compared to non-Tg mice. In lymph nodes, APP/PS1/Aβ-Th1 mice showed increased CD4+Tbet+ (*p* < 0.05) and CD4+RORγ+ T cells (*p* < 0.001) compared to APP/PS1 mice. Together, these results highlight the potential of Aβ-specific Teffs to contribute to exacerbation of a pro-inflammatory environment in AD progression.

### Aβ-Teffs affect Treg immunosuppression

**Fig. 5:**
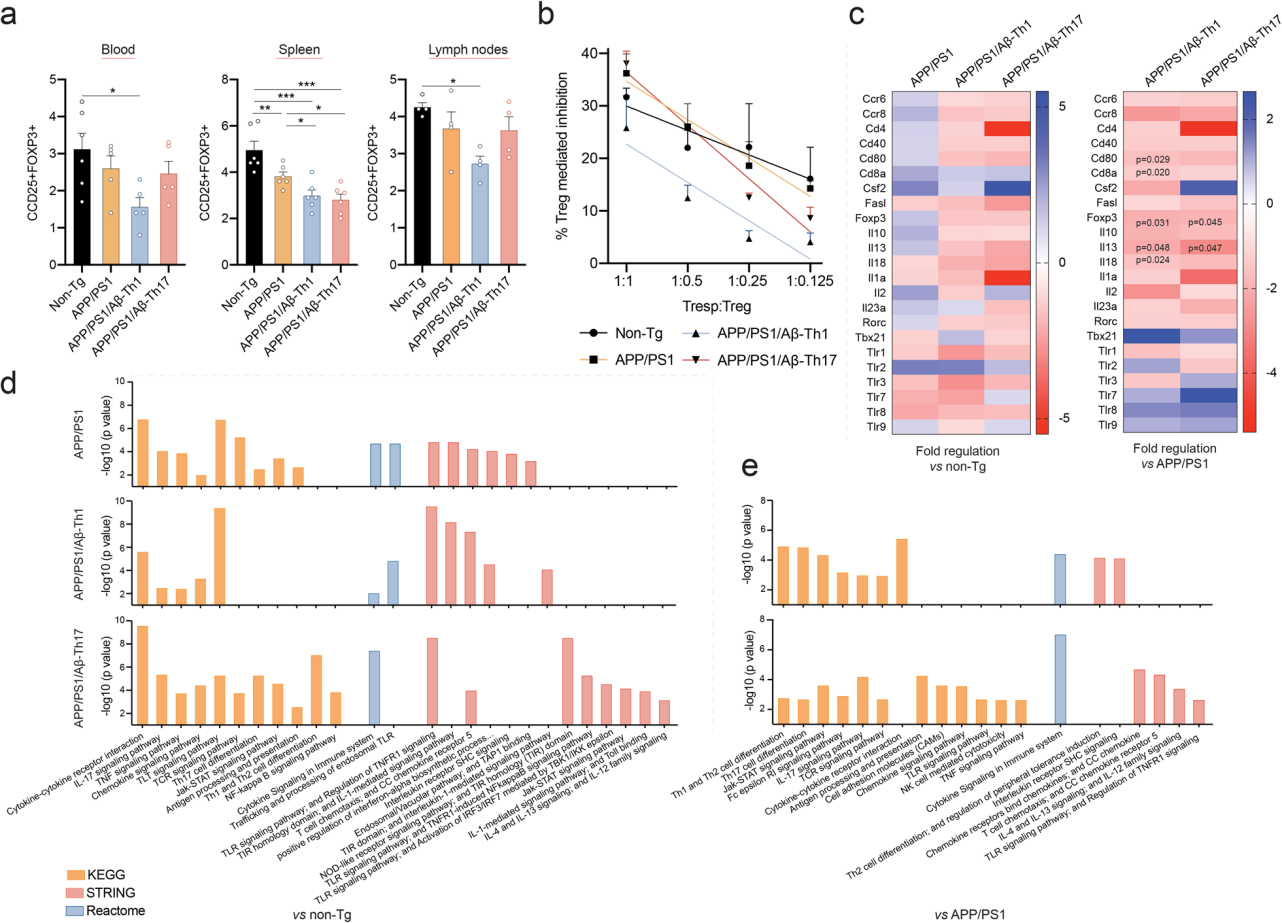
Aβ-Teffs affect Treg frequency and function. **a** Frequency of CD4+CD25+FOXP3+ Tregs in blood, spleen and lymph nodes from *n* = 6 mice per group. Statistical differences were determined using one-way ANOVA followed by Newman–Keuls post hoc test. *p < 0.05, **p < 0.01, ***p < 0.001, ****p < 0.0001. **b** Immunosuppressive function of Tregs assessed against proliferating CFSE-stained Tresps isolated from non-Tg mice. Tregs were isolated and pooled from within each group (*n* = 6 mice per group) and experiment was performed in triplicate. Linear regression analyses of Treg function from non-Tg mice showed *r*^2^ = 0.28 and *p* = 0.07. All other APP/PS1 groups showed *r*^2^ > 0.65 and *p* < 0.01. Although slops are not significantly different between different experimental groups, Th1 mice showed significantly different intercepts compared to non-Tg (*p* < 0.01) and APP/PS1 (*p* < 0.0001) mice. **c** Transcriptomic analyses for expression of innate and adaptive immune genes was performed using the RNA isolated from hippocampal tissues. Heat maps of fold changes in expression of genes compared to untreated or Aβ-Teff-treated APP/PS1 mice with non-Tg mice (left panel) and APP/PS1/Aβ-Th1 and APP/PS1/Aβ-Th17 mice compared to untreated APP/PS1 mice with significant *p* value provided in appropriate box (right panel). *n* = 4 mice per group analyzed using Qiagen RT^2^-PCR array. **d**, **e** Functional and pathway enrichment analysis of transcriptomic dataset was performed using KEGG, Reactome and STRING database. Different immune and inflammatory pathways affected in APP/PS1/Aβ-Th1 and APP/PS1/Aβ-Th17 mice were plotted as a bar chart in comparison to non-Tg (**d**) and untreated APP/PS1 mice (**e**). Significant pathways with *p* value passing Bonferroni-corrected significant level of 0.05 were plotted

Because afferent T cell and Teff responses modulate regulatory T cell (Treg) frequency and function [2, 34, 35], we assessed the effects of Aβ-Teffs on Tregs, which play important roles in the maintenance of immune tolerance against self- and non-self-antigens [2, 36]. Tregs suppress disease-reactive pro-inflammatory T cell responses via their unique transcription factor forkhead box P3 (FOXP3) [6, 37]. Treg frequencies were identified using surface and intracellular markers CD4+CD25+FOXP3+ in different lymphoid compartments. APP/PS1 mice showed decreased frequency of CD4+CD25+FOXP3+ cells (*p* < 0.01) in spleen compared to non-Tg mice (Fig. 5a). However, APP/PS1/Aβ-Th1 mice showed decreased CD4+CD25+FOXP3+ T cell frequency in blood (*p* < 0.05), spleen (*p* < 0.001) and lymph nodes (*p* < 0.05) compared to non-Tg mice, and in spleen (*p* < 0.05) compared to APP/PS1 mice. As Treg frequency does not always correlate with immunosuppressive function [38, 39], we sought to determine the effects of Aβ-Teffs on the Treg-mediated function. Early-stage APP/PS1 mice (5–6 months old) showed slightly increased Treg function (14%) compared to non-Tg mice at higher concentrations (1:1) which might be due to improved Treg function during early disease stages (Fig. 5b) [40]. However, APP/PS1/Aβ-Th1 mice showed significantly greater Treg dysfunction compared to non-Tg (slope *p* = 0.3650, intercept *p* = 0.002) and APP/PS1 (slope *p* = 0.9954, intercept *p* < 0.0001) control mice at all concentrations. Similar Treg dysfunction was observed in APP/PS1/Aβ-Th17 mice at lower concentrations compared to Tregs from non-Tg and untreated APP/PS1 mice. Overall, results indicated that Aβ-Th1 cells and to a lesser extent Aβ-Th17 cells diminished Treg frequency and function and suggests that diminution of Treg medicated control contributes to increased pro-inflammatory environment and on leads to the breakdown of immune tolerance [41].

### Aβ-Teffs induce Th1-dominant inflammatory responses in the CNS

We determined the effect of Aβ-Teffs on the expression of innate and adaptive immune genes within the CNS by using transcriptomic analysis of RNA from hippocampi of non-Tg and APP/PS1 mice treated with or without Aβ-Teffs. Gene expression of Treg transcription factor *Foxp3* (*p* < 0.05) and anti-inflammatory cytokines *Il10* and *Il13* (*p* < 0.05) and chemokine *Ccr8* were diminished in APP/PS1/Aβ-Th1 and APP/PS1/Aβ-Th17 mice hippocampi compared to untreated APP/PS1 mice (Fig. 5c), suggesting that adoptive transfer of Aβ-Teffs affect Tregs not only in the periphery, but also in the CNS. The inflammatory cytokine genes *Il5*, *Il6*, *Ifng* were decreased while *Tnf* was increased (1.43-fold) in APP/PS1/Aβ-Th1 compared to APP/PS1 mice (Additional file 1: Fig. S4). Importantly, Tbet encoding gene *Tbx21* expression increased in APP/PS1/Aβ-Th1 (2.59-fold) and APP/PS1/Aβ-Th17 (1.32-fold) compared against APP/PS1 mice. Notably, RORγ encoding gene *Rorc *expression decreased compared to non-Tg mice (Fig. 5c), underscoring predominate Th1 phenotype immune responses operative in the brains of Aβ-Teffs-treated APP/PS1 mice.

Adoptive transfer of Aβ-Teffs into APP/PS1 mice were studied for their abilities to induce changes in selected biological processes. To elucidate these, functional and pathway enrichment analyses were performed in mice treated with Aβ-Th1 or Aβ-Th17 cells then compared to non-Tg and APP/PS1 controls. Compared to non-Tg mice, Kyoto Encyclopedia of Genes and Gnomes (KEGG) analysis of APP/PS1, APP/PS1/Aβ-Th1 and APP/PS1/Aβ-Th17 mice demonstrated alterations in the cytokine–cytokine receptor interactions, IL17, TNF, chemokines, Toll-like receptor (TLR), Th17 cell differentiation, antigen processing-presentation, and Jak–STAT signaling pathways. There was greater enrichment of the TLR signaling pathway in APP/PS1/Aβ-Th1 mice. APP/PS1/Aβ-Th17 mice showed higher Th1, Th2, and Th17 cell differentiation pathways compared to APP/PS1 controls (Fig. 5d). These data, taken together, suggest that chronic CNS inflammatory responses are induced by Aβ-Th1 and Aβ-17 cells. APP/PS1/Aβ-Th17 mice also showed an enrichment of nuclear factor-kappa B (NF-κB) signaling pathway, which serves as a central mediator of inflammation because promoter regions of several pro-inflammatory molecules have been found to contain the DNA binding site for NF-κB [42]. Reactome analysis of different AD groups compared to non-Tg mice showed enrichment of cytokine signaling in immune system with higher induction in APP/PS1/Aβ-Th17 mice, while Search Tool for the Retrieval of Interacting Genes/Proteins (STRING) analysis showed enrichment of networks such as TLR signaling pathway; regulation of TNFR1 signaling, T cell chemotaxis; CC chemokine receptor 5, Toll/IL1 receptor homology (TIR) domain; and IL1-mediated signaling pathways with higher enrichment in APP/PS1/Aβ-Th1 mice compared to untreated APP/PS1 mice. Compared to untreated APP/PS1 mice, KEGG analysis showed enrichments of Th1, Th2, and Th17 cell differentiation, Jak–STAT, TCR, and cytokine–cytokine receptor pathways in APP/PS1/Aβ-Th1 mice, while IL17 signaling was enriched in APP/PS1/Aβ-Th17 mice (Fig. 5e). Th1, Th2 and Th17 cells are critical mediators of neuroinflammatory diseases and have been associated with the pathogenesis of several autoimmune diseases [43]. Reactome Gene Set Enrichment Analysis (GSEA) analysis showed enrichment of cytokine signaling in APP/PS1/Aβ-Th1 and APP/PS1/Aβ-Th17 mice compared to untreated APP/PS1 controls. These data indicated enhanced cytokine mediated pathogenesis in Aβ-Teff-treated mice. Additionally, STRING analysis showed enrichment of immune-related networks of interacting genes in Aβ-Th1 and Aβ-Th17-treated mice compared to untreated APP/PS1 mice. These data indicate that activated immune and inflammatory responses are exacerbated in Aβ-Teff adoptive transferred mice when compared to non-Tg and APP/PS1 controls.

### Aβ-Th1 cells facilitate amyloid deposition

**Fig. 6:**
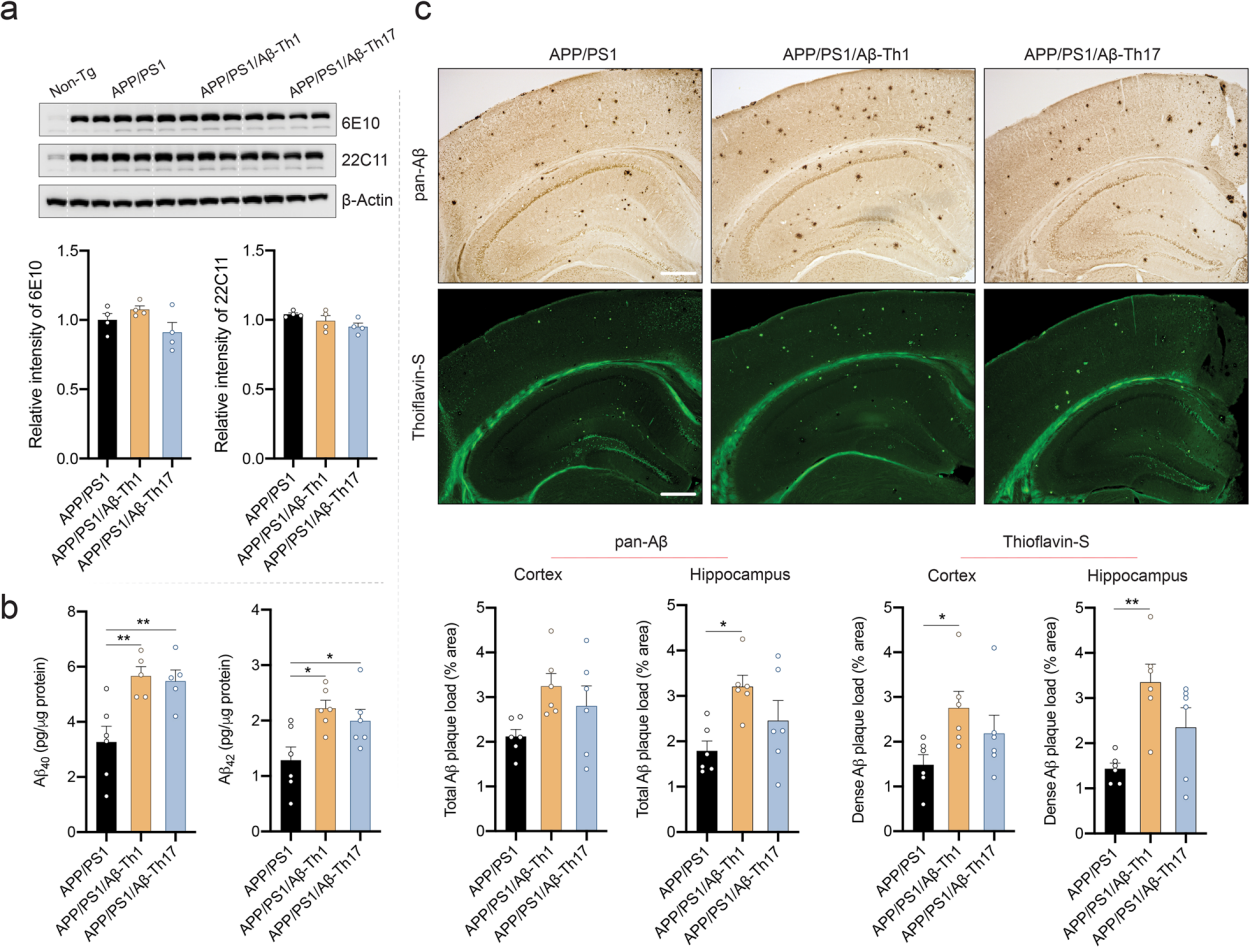
Aβ-Th1 and Aβ-Th17 cells increase amyloid load in APP/PS1 mice. **a** Western blot analysis performed to determine expression of full-length APP using cortical tissue lysate and 22C11 and 6E10 antibodies. Representative immunoblot and densitometric quantification was performed using different APP/PS1 mice. *n* = 4 mice per group were analyzed for statistical significance using one-way ANOVA followed by Newman–Keuls post hoc test. **b** ELISA performed to quantify Aβ_1–40_ and Aβ_1–42_ levels in the brain using Tris–HCl soluble fractions of cortical tissue. **c** Immunohistochemistry (pan-Aβ) and immunofluorescence (Thioflavin-S) performed to determine the area occupied by the insoluble Aβ plaque in the cortex and hippocampal brain regions. Representative images showing amyloid plaque DAB and Thioflavin-S staining in different brain regions. Percentage occupied area quantified using Cavalieri estimator probe of Stereo Investigator system (MBF Bioscience). Scale bar = 100 µm. *n* = 6 mice per group were analyzed. Statistical differences between groups determined using one-way ANOVA followed by Newman–Keuls post hoc test. *p < 0.05, **p < 0.01

Amyloid plaque forms upon sequential cleavage of APP attributed to different secretase enzyme activities. Before assessment of amyloid, we determined the effects of Aβ-Teffs on expression of full-length APP. Western blot analysis showed unaltered expression of full-length APP assessed by 6E10 and 22C11 in all APP/PS1 mice irrespective of Aβ-Teffs treatment (Fig. 6a). As amyloid plaque develops by aggregation of soluble Aβ oligomers, which are toxic and contribute to memory impairment [6], we next assessed the expression of soluble human Aβ_1–40_ and Aβ_1–42_ levels in cortical brain tissues from APP/PS1 mice. Aβ_1–40_ levels significantly increased in APP/PS1/Aβ-Th1 (*p* < 0.01) and APP/PS1/Aβ-Th17 (*p* < 0.01) mice compared to untreated APP/PS1 mice (Fig. 6b). Similarly, Aβ_1–42_ levels significantly increased in APP/PS1/Aβ-Th1 (*p* < 0.05) and APP/PS1/Aβ-Th17 (*p* < 0.05) mice compared to APP/PS1 mice. APP/PS1 mice develop amyloid plaque in the brain as early as 3 months of age [44], therefore we evaluated the effects of Aβ-Teffs on the amyloid plaque deposition in mice brain by immunohistochemistry which showed remarkable increases in amyloid plaque deposition in Aβ-Teffs-treated APP/PS1 mice (Fig. 6c). Indeed, total amyloid plaque loads increased in both cortex (53%) and hippocampus (*p* < 0.05) of APP/PS1/Aβ-Th1 mice compared to those of untreated APP/PS1 mice. APP/PS1/Aβ-Th17 mice showed total amyloid plaque loads 32% and 37% greater than APP/PS1 controls in cortex and hippocampus, respectively, but did not attain significance. The area occupied by the dense amyloid plaques was determined by Thioflavin-S staining of the same brain sections used for immunohistochemistry from different APP/PS1 mice. APP/PS1/Aβ-Th1 mice showed significantly increased dense amyloid plaque loads in cortex (*p* < 0.05, 86%) and hippocampus (*p* < 0.01, 134%) compared to APP/PS1 controls, while APP/PS1/Aβ-Th17 mice showed dense amyloid plaque loads 47% and 64% higher than APP/PS1 controls in cortex and hippocampus, respectively, but without significance. The results highlight that Aβ-Teffs drive amyloidosis in the APP/PS1 mice brain via aberrant APP cleavage without affecting APP production.

### Aβ-Th1 cells activate microglia

**Fig. 7:**
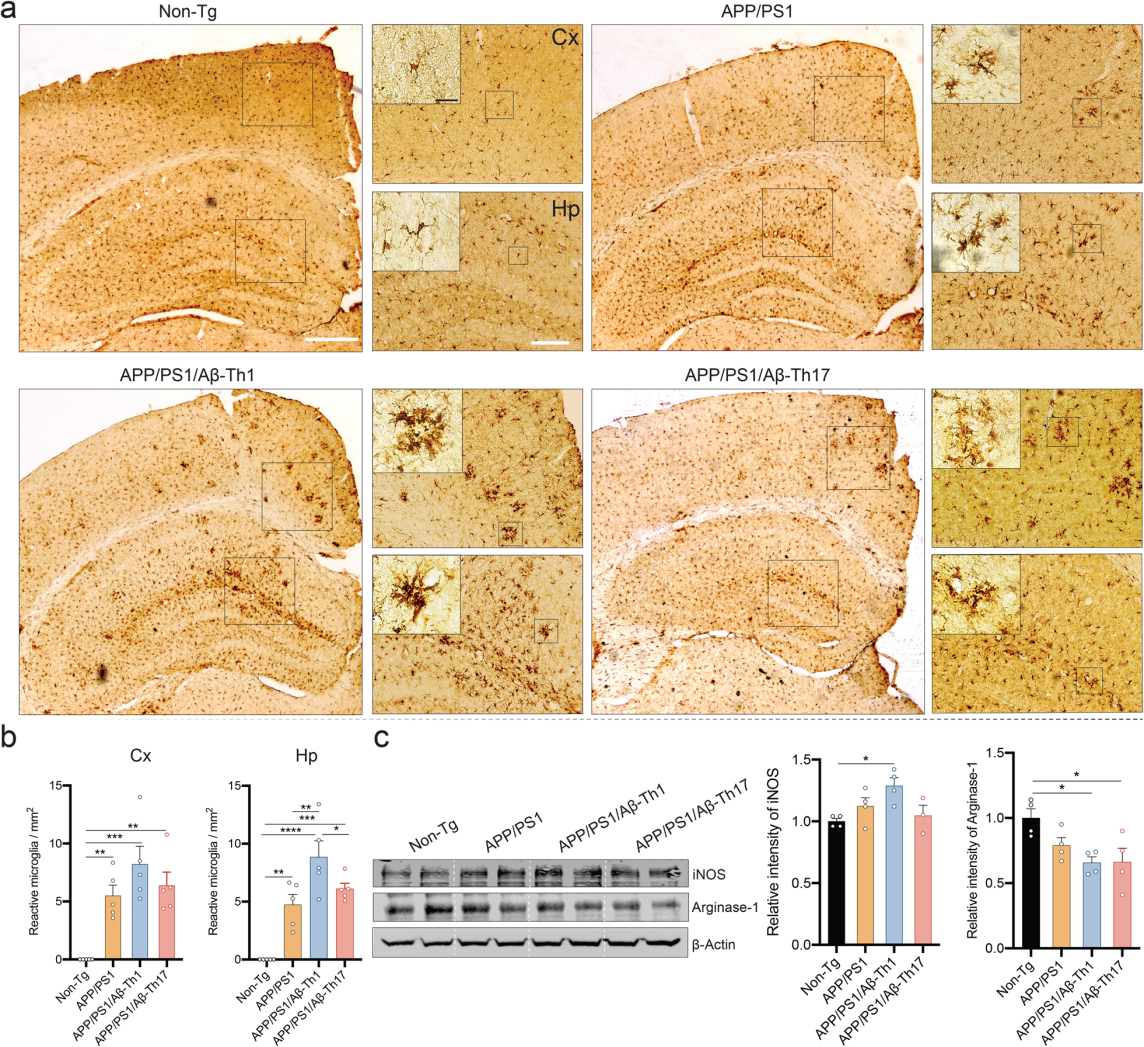
Aβ-Th1 and Aβ-Th17 cells activate microglia in APP/PS1 mice. **a** Immunohistochemistry performed to quantify number of reactive Iba1+ microglia cells in cortex and hippocampus brain regions. Representative pictures showing Iba1-reactive cells in different brain regions. Scale bar = 100 µm. Area with most Iba1-reactive cells highlighted by inserts for the cortex and hippocampus. Microglia morphology shown in higher magnification images. Scale bar = 20 µm. **b** Number of Iba1+-reactive microglia cells were quantified using optical fractionator module of Stereo Investigator system (MBF Bioscience). *n* = 5 mice per group were analyzed. **c** Western blot analysis performed to determine expression of iNOS and arginase-1 using cortical tissue lysate for assessment of microglial phenotypes. Representative immunoblot and densitometric quantification were performed using *n* = 4 mice per group. Statistical significance between different experimental groups determined using one-way ANOVA followed by Newman–Keuls post hoc test. *p < 0.05, **p < 0.01, ***p < 0.001, ****p < 0.0001

Microglia activation is a unique signature of neuroinflammation observed in AD patients and animal models [8], thus to determine the effects of Aβ-Teffs on microglial responses, we counted Iba1-reactive cells with amoeboid morphology in the cortex and hippocampus of different treatment groups. Immunohistochemistry visually showed that untreated APP/PS1 mice exhibited substantially more Iba1 positive (Iba1+) amoeboid cells compared to non-Tg mice suggesting microglial activation in early-stage AD mice (Fig. 7a). Moreover, remarkable numbers of reactive microglia were also observed in cortex and hippocampus of APP/PS1/Aβ-Th1 and APP/PS1/Aβ-Th17 mice. Stereological quantification revealed significantly increased numbers of Iba1+-reactive microglia in the cortex (*p* < 0.01) and hippocampus (*p* < 0.01) of untreated APP/PS1 mice brain compared to non-Tg mice (Fig. 7b). APP/PS1/Aβ-Th1 mice showed higher microglia activation in the cortex and hippocampus (*p* < 0.01) even compared to untreated APP/PS1 mice. APP/PS1/Aβ-Th17 mice also exhibited levels of reactive microglia in the cortex and hippocampus that were 16% and 29% higher, respectively, than untreated APP/PS1 mice, but did not attain statistical significance. To gain greater insights into microglia activation states, expression of the classical (M1) and alternative (M2) microglia activation markers inducible nitric oxide synthase (iNOS) and arginase-1, respectively [6, 9] was assessed in cortical tissues from experimental mice by western blot analysis. In early-stage disease of APP/PS1 mice, iNOS levels were elevated and arginase-1 levels diminished, though not statistically compared to non-Tg mice (Fig. 7c). However, APP/PS1/Aβ-Th1 mice showed increased production of iNOS, while adoptive transfer of Aβ-Th1 or Aβ-Th17 Teffs significantly reduced arginase-1 levels in recipient APP/PS1 mice (*p* < 0.05). These results revealed that Aβ-Teffs adoptive transfer potentiates progressive pro-inflammatory (M1) microglia activation status in APP/PS1 mice brain, wherein Aβ-Th1 cells are more capable of affecting microgliosis than Aβ-Th17 cells.

### Aβ-Th1 cells affect neuronal progenitor cell numbers and function

**Fig. 8:**
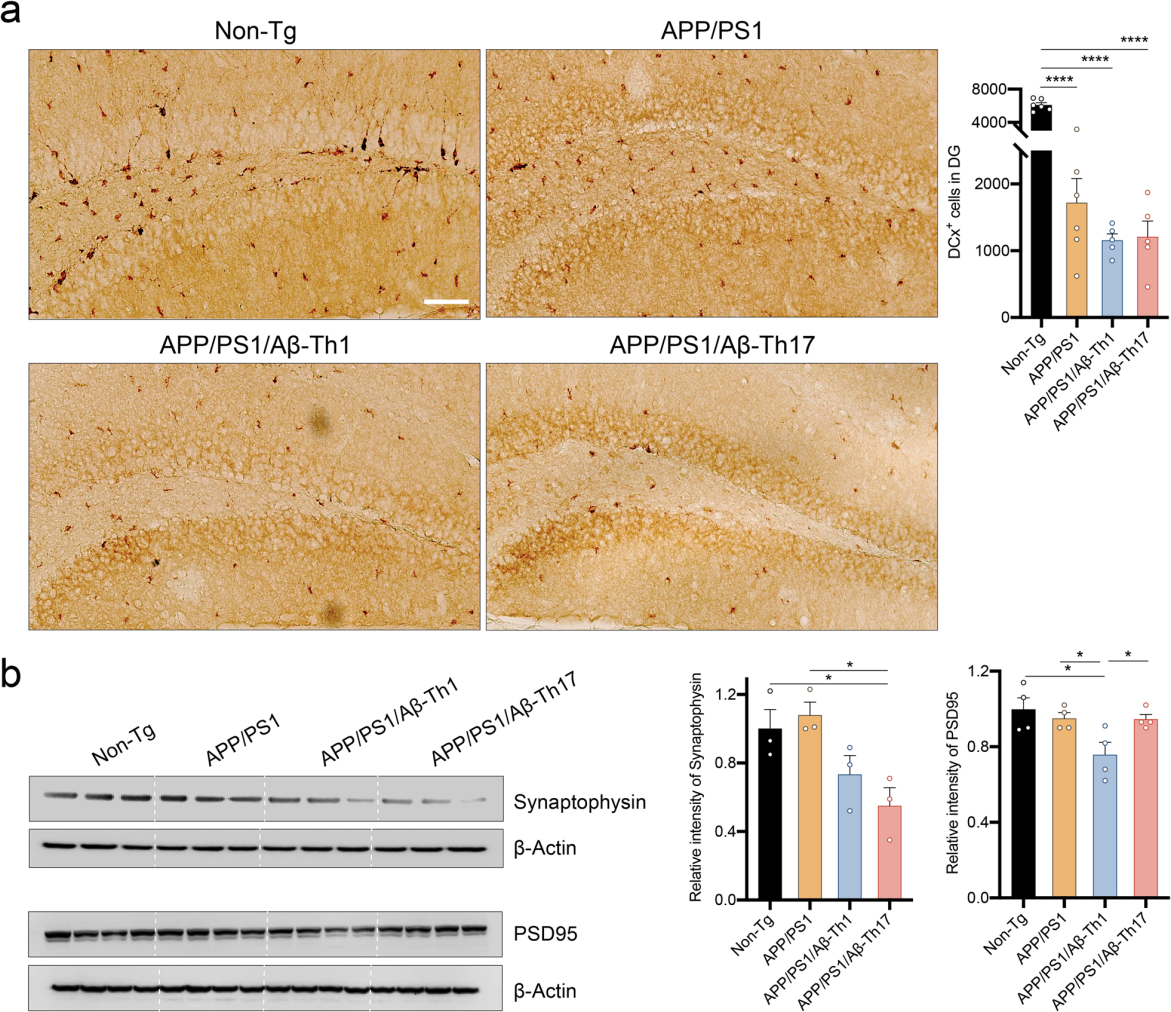
Aβ-Th1 and Aβ-Th17 cells affect neurogenesis and synaptic plasticity in APP/PS1 mice. **a** Neuronal progenitor cell density determined by immunohistochemical staining of doublecortin positive (Dcx+) cells in the dentate gyrus region of the hippocampus. Representative images showing Dcx+ cells. Scale bar = 20 µm. Dcx+ cells were quantified using the optical fractionator module of Stereo Investigator system (MBF Bioscience). *n* = 6 mice per group were analyzed. **b** Western blot analyses performed to determine expression of presynaptic (synaptophysin) and postsynaptic (PSD95) neurons using cortical tissue protein. Representative immunoblot and densitometric quantification performed for *n* = 4 mice per group. Statistical significance between groups determined using one-way ANOVA followed by Newman–Keuls post hoc test. *p < 0.05, ****p < 0.0001

Hippocampal neurogenesis correlates with memory function and neural plasticity; all affected during AD progression [45, 46]. To examine the effects of Aβ-Teffs on neurogenesis during AD progression, we assessed doublecortin (Dcx) as a biomarker of neuronal progenitors of the hippocampus [6, 45]. Dcx+ cell frequency was significantly diminished by 72% in the dentate gyrus of untreated APP/PS1 mice brain compared to non-Tg mice (*p* < 0.001, Fig. 8a). Adoptive transfer of Aβ-Teffs further reduced numbers of Dcx+ progenitors, compared to non-Tg controls (*p* < 0.001), but were not significant compared to APP/PS1 mice (32%). Next, we assessed expression of presynaptic (synaptophysin) and postsynaptic [postsynaptic density protein 95 (PSD95)] neurons in cortical brain lysates by western blot analysis. Co-expression of both pre- and post-synaptic proteins is essential for neuroplasticity, and therefore alteration of either protein level affects memory [6, 47]. Immunoblot quantification revealed that synaptophysin and PSD95 levels were unaltered in untreated APP/PS1 mice compared to non-Tg mice (Fig. 8b). However, expression of synaptophysin was reduced in APP/PS1/Aβ-Th1 (27% not significant) and APP/PS1/Aβ-Th17 (*p* < 0.05, 45%) mice compared to non-Tg mice. Additionally, APP/PS1/Aβ-Th1 (*p* < 0.05), but not APP/PS1/Aβ-Th17 mice showed reduced expression of PSD95 compared to non-Tg mice. Thus, only presynaptic protein abnormalities were observed in APP/PS1/Aβ-Th17 mice, while pre-and post-synaptic protein densities were affected in APP/PS1/Aβ-Th1 mice. Overall, Aβ-Th1 and Aβ-Th17 cell-mediated exacerbation of neuronal synaptic disintegrity in amyloid-rich environments which contribute to Aβ-Teffs’ potential to accelerate memory impairment in APP/PS1 mice.

## Discussion

Previous studies demonstrated a spectrum of effects for Aβ-reactive Teffs in animal models and AD patients [17–19, 48, 49]. However, one of the limitations of those studies is that polyclonal Teffs used were short-term maintained (2 to 4 weeks), unstable, and prone to plasticity, which are not ideal for the study of T cell processes in AD and their underlying complex mechanism of actions. The polyclonal cells, unlike cloned populations, contain mixtures of several putative, but unexpressed phenotypes, which could overwhelm the population during the short culture time or possibly be eliminated. Therefore, we generated stable, long-lived Aβ-Th1 and Aβ-Th17 Teff clones, which were maintained for more than 6 months without compromising cytokine signatures and antigen specificities prior to use in in vivo experiments. Adoptive transfer of Aβ-Th1 and Aβ-Th17 Teffs exacerbated memory impairment and amyloid deposition in APP/PS1 mice. The results vary with a previous report where only IFNγ-secreting Aβ-Th1 cells produced detrimental memory effects in APP/PS1 mice [18]. One possible explanation is that instead of independent development, we polarized Aβ-Th1 cells into Aβ-Th17 Teffs using selective culture media. Adoptive transfer of Aβ-Th17 cells may have induced a combined Th1/Th17 Teff pathogenesis via IFNγ and IL12 signaling [50]. In agreement with our results, clinical studies have shown increased frequency of IFNγ secreting Th1 [51] and IL17-secreting Th17 cells [14, 15] in the early-stage AD patients compared to healthy controls. Our own and other laboratories have demonstrated neurotoxic effects of antigen-specific Th17 cells in PD animal models [34] and patients [39]. Indeed, peripheral adaptive immune impairments are comparable between the two most common neurodegenerative diseases, AD and PD [35, 39, 52], and has been speculated that disease operative Th17 cells are also deleterious, although to a lesser extent than Th1 cells in AD pathogenesis.

Systemic inflammation is operative in AD patients before the onset of memory impairment. This suggests an important role of peripheral immune activation in disease [15, 32, 33]. Aβ drains to the peripheral cervical lymph nodes [53] where APCs process and present Aβ peptides to peripheral T cells engaging MHC-I or -II, which initiates peripheral adaptive immune activation [2, 11, 54]. However, with disease progression, Aβ lymphatic drainage is compromised leads to the formation of cerebral amyloid angiopathy and Aβ accumulation [55]. In parallel, clonally expanded and activated peripheral T cells expressing chemokines (CCL3, CCL4) cross the blood–brain barrier (BBB) and affect resident microglia and neuronal cells perpetuating a neuroinflammatory state [21, 56]. Recently, Gate et al. showed elevated frequency of CD8+ effector memory T cells in the blood of AD patients which negatively correlated with memory function [13]. The peripheral CD8+ Teffs invaded the CNS, clonally expanded into the cerebrospinal fluid, and contributed to neuroinflammation [13]. Here, adoptive transfer of Aβ-Th1 and Aβ-Th17 Teffs into APP/PS1 mice increased the frequency of antigen-specific T cells. This was demonstrated by increased expression of TNFα-, IFNγ- and IL17-secreting and MHCII-IA^b^-Aβ peptide tetramer reactive CD4+ T cells. Elevated Th1 and Th17 cytokines then weaken tight junctions of the BBB to allow peripheral inflammatory mediators, including TNFα, IL1β, and IL6, entrance into the brain serving to further activate resident microglia. We contend that reactive microglia contribute to aberrant adaptive immune responses by presenting cognate antigen and secreting pro-inflammatory cytokines [57]. The direct involvement of Aβ-Teff-induced systemic inflammatory responses in the neuroinflammatory cascade and amyloid deposition is a pivotal pathogenic process for AD.

Previous studies reported decreased frequency and function of anti-inflammatory and immunosuppressive CD4+CD25+FOXP3+ Tregs in blood of AD patients [52, 58] and experimental animal models [6]. Additionally, transient breaking of immune tolerance by Treg depletion is associated with amyloid clearance and neuroinflammation restoration via subsequent CNS recruitment of immunoregulatory Treg and monocyte-derived macrophages [41]. Other reports demonstrated the neuroprotective potential of polyclonal Treg-adoptive transfer or expansion in AD animal models [59, 60], PD [61, 62], and ALS patients [63]. Together, these reports suggest a role of Treg deficits in AD progression, but the underlying cause is unknown. For the first time, we identified the role of disease operative CD4+ Aβ-Teff subsets in Treg dysfunction in APP/PS1 mice. We demonstrated that adoptive transfer of Aβ-Th1 and Aβ-Th17 cells significantly reduced the frequency of CD4+CD25+FOXP3+ Tregs in blood, spleen and lymph nodes of APP/PS1 mice. Additionally, Treg immunosuppressive function was greatly compromised in APP/PS1 mice receiving Aβ-Th1 cells compared to Aβ-Th17 treated or untreated APP/PS1 mice. Interestingly, Aβ-Teffs affected Treg function in both periphery and CNS. It is speculated that antigen-specific CD4+ T cells detected after adoptive transfer elicited Treg impairments to progress AD pathology in mice [2, 64].

Microglia serve key roles in processing and presenting self-antigens including Aβ to T cells to maintain immune tolerance [2, 19]. Non-activated microglia exhibit ramified morphology and are able to clear Aβ deposits through phagocytosis. However, with disease progression, microglia become more activated and acquire amoeboid morphology with compromised phagocytic capabilities [9]. Upon antigenic stimulation, microglia can acquire either classically activated (M1) or alternatively activated (M2) phenotype, representing pro-inflammatory and anti-inflammatory phenotypes, respectively [6, 9]. Here, we identified that expression of iNOS, an M1 marker, was significantly elevated while that of arginase, an M2 marker, was reduced in the brain of APP/PS1/Aβ-Th1 mice, suggesting that disease-linked CD4+ Teffs alter microglial polarization leading to worsen disease outcomes. Recently, using genome-wide analysis and single-cell mass cytometry [65, 66], different microglial clusters were identified in various brain regions of humans and mice and were found to predominantly exist distinctly to the physiological and pathological conditions. However, the unique function associated with each microglia subset has not yet been identified. These reports suggest that microglia phenotypes are much more complicated and beyond simplified M1 and M2 phenotypes [67, 68]. Functional and pathway enrichment analysis revealed that Th1, Th2 and Th17, and cytokine and TLR signaling pathways are enriched in APP/PS1 mice brain following adoptive transfer of Aβ-Teffs further confirming the ability of Aβ-Teffs to perpetuate immune and neuroinflammatory responses.

In addition to affecting microglia responses, Aβ-Th1 cells significantly increased amyloid deposition in both cortex and hippocampus regions of brain as demonstrated by increased soluble Aβ_1–40_ and Aβ_1–42_ as well as insoluble Aβ plaques without affecting full-length APP production. Aβ-Th17 cell treatment affected amyloid load and microglial responses in APP/PS1 mice brain to a lesser extent compared to Aβ-Th1 cells which might be due to their less extensive inflammatory responses as those induced by Aβ-Th1 cells in an amyloid-rich environment. The results were further supported by the previous study showing Th1 biased immune responses accountable for most of the CD4+ Teffs’ detrimental effects in APP/PS1 mice [18]. Neuroinflammation arose either as a result of Teff-driven microglia responses or through direct cytokine release [2, 11] affecting synaptic integrity and neurogenesis. These are both hallmarks of neurodegeneration [6, 9]. Functional co-localization of pre- and post-synaptic neurons is essential for memory development, whereby defects in either neuron can worsen memory function [9, 47]. Adoptive transfer of Aβ-Th1 and Aβ-Th17 cells showed significantly defective post-synaptic protein expression in APP/PS1 mice. Neuronal progenitor development is affected in the dentate gyrus region of APP/PS1 mice, which was further worsened by Aβ-Teffs, suggesting Aβ-Teffs impose a detrimental role in neurogenesis.

A previous study demonstrated that complete loss of peripheral adaptive immune system exacerbated amyloid deposition and neuroinflammation in 5XFAD-Rag mice and that reconstitution of this cell population restores AD pathology [7]. Likewise, from HIV infected individuals, 50% of patients develop AD-linked memory deficits later in life despite antiretroviral therapy, in part due to selective functional decline of peripheral CD4+ T cells [69]. Although, adaptive immune T cells are essential to control AD pathology, Aβ-reactive Teffs can develop meningoencephalitis as evidenced in a Phase IIa clinical trial (NCT00021723) with full-length Aβ immunization in QS21 adjuvant [48, 49]. Indeed, considering studies of the CNS effects of Aβ-Teffs in AD patients and animal models [19, 49], ours is the first study characterizing effects of stable, long-lived Aβ-Th1 and Aβ-Th17 Teff clones on neuroinflammation in the AD mouse model. We identified that Aβ-Th1 cells induced consistent pro-inflammatory responses in both periphery and CNS leading to memory impairment, increased amyloid load, microgliosis, and impaired neurogenesis. Tregs were identified as a key regulator of Aβ-Teff-driven neuroinflammation in amyloid-rich environment leading to the breakdown of immune tolerance [2, 41]. Thus, we postulate that development of Aβ-specific Tregs, instead of polyclonal Tregs associated with the risk of global immunosuppression [70] might better serve as a safe and potential therapeutic alternate for management of elderly AD patients.

## Conclusions

Disease-reactive Aβ-Th1 and Aβ-Th17 cells transform a pro-inflammatory microenvironment to accelerate AD pathology in APP/PS1 mice. The detrimental effects of Aβ-Teffs are sped by limiting Treg activities in the periphery and CNS. Control of this neurodestructive environment represents a potential therapeutic strategy and can be sped by augmenting peripheral Treg numbers and function reactive against disease-specific protein.

## Methods

### Development of Aβ-Th1 and Aβ-Th17 cells

Six-month-old, two female B6;129 mice were immunized by subcutaneous injection with human Aβ_1–42_ (50 µg) (Catalog no. 03-112, Life Technologies) emulsified in complete Freund’s adjuvant (Sigma Aldrich) containing *Mycobacterium tuberculosis* (1 mg/ml). The mice were boosted after 2 weeks with Aβ_1–42_ (50 µg) in incomplete Freund’s adjuvant (Sigma Aldrich). One week after the second immunization, the mice were killed, and spleen and lymph nodes (axial, brachial, cervical, and inguinal) were harvested then mashed through 70-μm cell strainer to prepare single cell suspensions. The red blood cells were lysed with ammonium–chloride–potassium (ACK) lysis buffer (Catalog no. A10492, Thermo Fisher Scientific). CD4+ T cells were enriched from single cell suspensions using an EasySep™ mouse CD4+ T cell isolation kit (Catalog No. 19852, Stemcell Technologies) and recovered CD4+ T cells (98% CD4+ by flow cytometric analysis) were cultured in vitro in the presence of monomeric Aβ_1–42_ (25 μg/ml) prepared as described before [71] (Catalog no. RP10017, GenScript) and 2.5 × 10^7^ feeder cells, i.e., splenocytes isolated from naïve female B6;129 mice and irradiated at 3200 Gy (RS 2000 Irradiator, Rad Source, Buford, GA). Cells were propagated in RPMI-1640 media supplemented with 10% fetal bovine serum (FBS), 2 mM L-glutamine, 25 mM HEPES, 1 mM sodium pyruvate, 1× nonessential amino acids, 55 nM 2-mercaptoethanol, 100 U/ml penicillin, and 100 µg streptomycin. Fresh culture media, Aβ, and feeder cells were provided weekly and the viable CD4+ T cells were counted before each passage. CD4+ T cells non-reactive to Aβ were eliminated over passages as evidenced by declined viable CD4+ cell count up to day 21 (Additional file 1: Fig. S1a). On day 28, increased Aβ-reactive CD4+ T cell count was observed and at this point 25 U/ml interleukin-2 (IL2) was added to the culture, leading to further increase in Aβ-reactive CD4+ count thereafter. To recover CD4+ T cell clones, cells were cultured at 1 cell/well in flat-bottom 96-well plate in presence of fresh feeder cells and Aβ_1–42_. The most rapidly growing Aβ-reactive clone was identified and propagated with IL2, Aβ_1–42_, and feeder cells, and hereafter is designated as Aβ-Th1 cells. A subclone of Aβ-Th1 was polarized to Th17-type Teffs by culturing in complete RPMI media supplemented with 3 ng/ml TGFβ, 25 ng/ml IL6, 5 ng/ml IL1β, 20 ng/ml IL23 and 3 µg/ml of antibodies to IL4, IFNγ, and IL2 in the presence of Aβ_1–42_ and fresh feeder cells; hereafter designated as Aβ-Th17 cells.

### Cell phenotypes and antigen-specificities

To induce cytokine production, 10^6^ Aβ-Th1 or Aβ-Th17 cells were stimulated in complete RPMI-1640 media containing 20 ng/ml PMA, 1 µM ionomycin (Sigma Aldrich), and 3 µg/ml brefeldin A (Catalog no. 4506521, eBioscience) for 12 h, then extracellular/intracellular staining was performed for flow cytometric analysis as described before [6, 72]. Cells were first stained for surface biomarker expression with anti-CD3e-PE (Catalog no. 12003181, eBioscience), anti-CD4-APC-H7 (Catalog no. 560181, BD Pharmingen), and anti-CD8a-PE-Cyanine 5.5 (Catalog no. 35008180, eBioscience) antibodies for 30 min at room temperature. For intracellular staining, cells were fixed and permeabilized using transcription factor staining buffer kit (Catalog no. 552300, eBioscience) for 45 min at 4 °C, followed by incubation with anti-IFNγ-FITC (Catalog no. 11731182, eBioscience), anti-TNFα-eFluor 450 (Catalog no. 48732182, eBioscience), anti-IL17a-Alexa Fluor 647 (Catalog no. 506912, BioLegend), anti-Tbet-eFluor 660 (Catalog no. 50582582, eBioscience) and anti-RORγ-PerCp eFluor 710 antibodies (Catalog no. 46698182, eBioscience) for 30 min at 4 °C. Isotype and fluorescence-minus-one (FMO) controls for each antibody were used during flow cytometric analysis for accurate gating of different cell subsets. For analysis of extracellular cytokine release, Aβ-Th1 and Aβ-Th17 cells were stimulated with 20 ng/ml PMA and 1 µM ionomycin for 12 h and culture supernatants were analyzed using proteome profiler mouse cytokine array kit (Catalog no. ARY006, R&D Systems) according to the manufacturer’s instructions. CD3+CD4+ T cells were gated to quantify the intracellular cytokine and transcription factor expression. Few endogenous feeder cells were present during extracellular immunoblot staining that might have affected the cellular cytokine signature during short activation period.

From Aβ_1–42_ sequence, 1 to 15 amino acid region (DAEFRHDSGYEVHHQ) comprises B cell epitope while 15 to 30 amino acid region (KLVFFAEDVGSNKGA) comprises T cell epitope in the mice while in human it may be up to 15 to 42 amino acid region [51]. To elucidate the ability of Aβ-Th1 and Aβ-Th17 cells to recognize the T cell epitope of Aβ_1–42_ presented by H-2^b^ haplotypes (feeder cell matched), tetramers were constructed with I-A^b^ and the amino acid 15–30 (MHCII-IA^b^–KLVFFAEDVGSNKGA) conjugated to fluorophore BV421 (National Institute of Health (NIH) Tetramer Core Facility, Emory University, Atlanta, GA). MHCII-IA^b^–PVSKMRMATPLLMQA tetramer with no Aβ specificity was used as control. For tetramer staining, 3 × 10^5^ Aβ-Th1 or Aβ-Th17 cells were incubated with increased concentrations of MHCII-IA^b^–KLVFFAEDVGSNKGA Aβ tetramer (1.2 µg, 2.4 µg and 12 µg) or MHCII-IA^b^–PVSKMRMATPLLMQA control tetramer (2.4 µg) for 3 h at 37 °C. After incubation, tetramer-stained T cells were reacted with anti-CD3e-PE and anti-CD4-APC-H7 antibodies for 30 min at room temperature, followed by live-dead staining with propidium iodide (0.5 µg/ml) for 5 min at room temperature. Stained T cells were analyzed with a LSR II flow cytometer and FACSDiva Software (BD Bioscience) at the University of Nebraska Medical Center Flow Cytometry Research Facility.

### T cell receptor identification

Total RNA was isolated from the parent T cell clone (Aβ-Th1 cells) using RNAeasy mini kit (Catalog no. 74104, Qiagen). First strand complementary DNA (cDNA) was synthesized and TCRα and TCRβ chain variable region sequences were amplified using SMARTer^®^ mouse TCR a/b profiling kit (Catalog no. 634402, Takara). For the TCR sequence identification, PCR products generated were cloned into the pCR™ 4Blunt-TOPO^®^ plasmid using Transform One Shot^®^ Mach-T1™ competent cells following manufacturer’s instructions (Catalog no. 450031, Life Technologies). Bacterial colonies were grown in LB agar media followed by DNA isolation and clean-up using monarch plasmid miniprep kit (Catalog no. T1010S, New England BioLabs) and samples were submitted for Sanger sequencing with M13 primers at the University of Nebraska Medical Center Genomics Core Facility.

### Modeling of TCR–pMHC complex

The TCR-Aβ–MHCII (TCR–pMHC) complex was constructed with the fully automated structure preparation and homology modeling in the Schrodinger BioLuminate suite [73]. The loops were modeled using ab initio loop modeling and cross validated on the SwissModel server [74, 75]. The quality estimation of the generated model and subsequent refinements were performed by MolProbity and QMEAN method on SwissModel [76]. The full query sequence was used for the modeling studies, MHC consisted of two chains: MHCII-IA^b^α with 256 residues (1–256) and MHCII-IA^b^β with 264 residues (257–521). Aβ peptide had 42 residues (522–563), TCRα consisted of 274 residues (564–838) and TCRβ consisted of 305 residues (839–1144). The MHC–peptide residues were first modeled with the antibody modeling tools prediction and advanced homology modeling. The TCRα/β complex was modeled later and both models were analyzed separately for structural features. The single model was generated from the above-mentioned tools, the structural features were validated from the previously generated models. *System preparation*: All molecular dynamics (MD) simulations were performed on AMBER 18 software package [77]. The protein complex was prepared with the help of XLeap [78]. The TCR–pMHC complex was solvated in a truncated octahedron box unit of dimensions *x* = 192.98, *y* = 186.34 and *z* = 182.65. Total of 125,812 TIP3P water molecules were added to solvate the system [79]. A sufficient number of counter ions, Na+ and Cl−, were added to neutralize the simulation system and achieve 0.14 M of ionic strength. FF14SB force field was used to parameterize the amino acids and to model the proteins [80]. *Unbiased explicit solvent MD simulation*: Simulations were performed for 100 ns of time step on Nvidia V100-SXM2-16 GB Graphic Processing Unit using the PMEMD.CUDA module [81]. Simulations were run at 1 atm constant pressure using Monte Carlo barostat and 300 K constant temperature by using Langevin thermostat with a collision frequency of 2 ps-1 and the volume exchange was attempted for every 100 fs. An integration step of 2 fs was also used for simulation of hydrogen atoms involving bonds were constrained by using SHAKE algorithm [82]. Long-range electrostatic interactions were computed using Particle Mesh Ewald method while for short-range interaction a cutoff of 8 Å was used [83]. Equilibration consisted of rounds of NVT and NPT equilibration for 10 ns in total. CPPTRAJ [84] was used to analyze the interactions over full trajectory after taking configuration at every 4 ps. RMSD, RMSF was determined after analyzing the trajectories.

### Adoptive transfer in APP/PS1 mice

All animal experiments were approved by the institutional Animal Care and Use Committee of University of Nebraska Medical Center. Transgenic mice overexpressing human APP695 with the Swedish mutation (Tg2576) were obtained from Drs. G. Carlson and K. Hsiao-Ashe through the Mayo Medical Venture [85]. PS1 mice overexpressing human PS1 with M146L mutation were provided by Dr. K. Duff through the University of South Florida [86]. Both mice were maintained on the B6;129 hybrid background. Male Tg2576 mice were crossbred with female PS1 mice to generate APP/PS1 double-transgenic mice and non-transgenic (non-Tg) B6;129 mice were developed in parallel as described previously [6, 9, 87, 88]. Female APP/PS1 mice, 4–5 months old, and age-matched non-Tg littermates were randomly divided into the different experimental groups. Either 1 × 10^6^ Aβ-Th1 or Aβ-Th17 cells in 100 µl phosphate-buffered saline (PBS) were adoptively transferred to the APP/PS1 recipient mice, intravenously via tail vein using a 28-gauge needle affixed to a sterile tuberculin syringe, twice at 1-week intervals. Untreated and age-matched non-Tg mice served as control.

### Radial arm water maze test

Two weeks after the second adoptive cell transfer, mice were submitted for radial arm water maze (RAWM) testing in a blinded fashion to assess memory impairment as previously described [6, 71]. Briefly, mice from masked cages were introduced into the circular water filled tank (diameter-110 cm and height-91 cm, San Diego Instruments) with triangular inserts that produce six swim paths radiating from the center. Special cues are fixed on the tank wall to guide mouse orientation. At the end of any one arm, a circular Plexiglass hidden platform (diameter-10 cm) is placed submerged 1 cm beneath the water level. The platform was placed in the same arm for four consecutive acquisition trials (T1–T4), and retention trial (T5), but in a different arm on different experimental days. For T1–T4, the mouse started the task from a randomly chosen arm without a platform. After four trials, the mouse was returned to its cage for 30 min and reintroduced into the T4 arm, for the delayed retention trial (T5). Each trial lasted 1 min, and an error was scored when mouse entered the wrong arm; entered the arm with the platform, but did not climb on it; or did not make a choice for 20 s. The trial ended when the mouse climbed and stay on the platform for at least 10 s. The mouse allowed to rest on the platform for 20 s between trials. If the mouse did not climb the platform, after 60 s it was gently guided to the submerged platform. The T1, T4 and T5 trial errors over 9-day test were divided into three blocks (block-1 days 1–3, block-2 days 4–6, block-3 days 7–9), and the errors in each block were averaged for statistical analysis.

### 2-Deoxy glucose chemical exchange saturation transfer (glucoCEST) MRI

Mice were fasted for 24 h and fasting blood glucose concentrations were measured prior to the experiment using glucometer and test strips (ReliOn™ Prime) by collecting blood from tail vein puncture. Mice were anesthetized with isoflurane in the mixture of oxygen and the peritoneal cavity was cannulated for the injection of 2-deoxy glucose (2DG). Cannulated mice were fixed on a ^1^H magnetic resonance imaging (MRI)-compatible cradle using a bite bar. MRI was performed on a 7-Tesla scanner (Bruker PharmaScan, Billerica, MA) with a Bruker-built quadrature mouse brain RF coil. Respiration and body temperature were monitored during scanning. A baseline glucose CEST (glucoCEST) MRI was acquired followed by 2DG (1 g/kg in PBS) injection via intraperitoneal catheter into the mice to monitor glucose signal in the brain over the time. GlucoCEST data were acquired using a rapid acquisition with relaxation enhancement (RARE) sequence (repetition time (TR)/echo time (TE) = 1600/16 ms, RARE factor = 8) with a continuous radio frequency (RF) for saturation with the power = 3 µT, duration = 1 s, saturation frequencies = − 1600 to 1600 Hz in steps of 80 Hz. A second CEST data with saturation RF power = 0.5 μT, and frequencies = − 300 to + 300 Hz were acquired for B0 inhomogeneity correction using WASSR [89]. The glucoCEST scan time was ~ 10 min, and was repeated at 10, 20, 30, 40, 50, and 60 min after 2DG injection. Asymmetric magnetization transfer ratio (MTRasym) was calculated from the Z-spectrum that was built based on CEST data. The glucoCEST signal was calculated as the integral of the MTRasym within 1.00 ± 0.25 p.p.m.

### Flow cytometry

On day 42, mice were terminally anesthetized with pentobarbital followed by spleen isolation in complete RPMI media and blood collection via cardiac puncture in the K3EDTA tubes (Catalog no. 450475, Greiner BioOne North America). Lastly, mice were pericardially perfused with PBS and lymph nodes (axial, brachial, cervical, and inguinal) and brain were harvested. Whole blood was stained while single cell suspension was prepared from spleen and lymph nodes. Either 50 μl of blood or 1 × 10^6^ spleen or lymph node cells were stained for flow cytometric analysis as described above using antibodies: anti-CD3e-PE, anti-CD4-APC-H7, anti-CD8a-PE-Cyanine 5.5, anti-CD25-PE-Cy7 (Catalog no. 25025182, eBioscience), anti-Tbet-eFluor 660, anti-RORγ-PerCp eFluor 710 and anti-FOXP3-Alexa Fluor 488 (Catalog no. 320012, BioLegend). For intracellular cytokine analysis, 1 × 10^6^ spleen cells were stimulated with 20 ng/ml PMA and 1 µM ionomycin in presence of brefeldin A for 12 h and after incubation cells were stained with anti-CD3e-PE, anti-CD4-APC-H7, anti-IFNγ-FITC, anti-TNFα-eFluor 450, and anti-IL17a-Alexa Fluor 647 antibodies. To determine the frequency of Aβ reactive CD4+ T cells, 1 × 10^6^ lymph node cells were stimulated with Aβ_1–42_ (25 μg/ml) in presence of feeder cells and IL2 (25 U/ml) for 5 days at 37 °C. On day 5, cells were collected by centrifugation and incubated with MHCII-IA^b^–KLVFFAEDVGSNKGA Aβ tetramer (6 µg) or MHCII-IA^b^–PVSKMRMATPLLMQA control tetramer (6 µg) for 3 h at 37 °C. After incubation, T cell-MHCII-Aβ tetramer complex was stained with anti-CD3e-PE, anti-CD4-APC-H7 antibodies and propidium iodide for flow cytometric analysis.

### Treg function assay

CD4+CD25+ Tregs and CD4+CD25− T responder cells (Tresps) were isolated from the mice spleen as described earlier [72] using EasySep™ mouse Treg enrichment kit (Catalog no. 18783, Stemcell Technologies) as per the manufacturer’s instructions. Briefly, CD4+ T cells from single cell suspensions were first enriched by negative selection using the EasySep™ mouse CD4+ T cell isolation cocktail from which CD25+ cells were then positively selected using EasySep™ mouse CD25+ Treg selection cocktail. The isolated CD4+CD25+ cells were more than 97% FOXP3+ as determined by flow cytometric analysis. CD4+CD25− Tresps, more than 96% pure, were collected from naïve non-Tg mice spleens and used in the proliferation assay after labeling with carboxyfluorescein succinimidyl ester (CFSE) (Catalog no. C34554, Thermo Fisher Scientific). CD4+CD25+ Tregs from different experimental groups were serially diluted in U-bottom 96-well plate to obtain 50, 25, 12.5, and 6.25 × 10^3^ Tregs in 100 µl of media followed by addition of 50 × 10^3^ CFSE-labeled Tresps into each well to obtain Tresp:Treg rations of 1:1, 1:0.5, 1:0.25 and 1:0.125, while wells with only Tresps served as controls. Mouse T cell activating CD3/CD28 Dynabeads (Catalog no. 11456D, Thermo Fisher Scientific) were added to each well at a bead:Tresp ration of 1:1 to induce Tresp proliferation. The immunosuppressive function of Tregs to inhibit proliferation of CFSE-stained Tresps was determined after 72 h incubation at 37 °C using flow cytometric analysis and is reported as Treg-mediated % inhibition.

### Transcriptomic and functional and pathway enrichment analysis

Hippocampal tissue was isolated from mouse brain and total RNA extracted with the RNAeasy mini kit (Catalog no. 74104, Qiagen). Recovered RNA was reverse transcribed into cDNA using a RevertAid First Strand cDNA Synthesis kit (Catalog no. K1622, Thermo Fisher Scientific). One microgram cDNA was amplified using primer mix from the RT^2^-PCR array for Mouse Innate and Adaptive Immune Responses (Catalog no. 330231, Qiagen). Quantitative RT-PCR was performed using Mastercycler Realplex EP (Eppendorf) and data were analyzed using RT^2^ Profiler PCR array web-based data analysis software (Qiagen). Next, we conducted functional and pathway enrichment analysis of genes deregulated in APP/PS1 mice untreated or treated with Aβ-Teffs compared to non-Tg and APP/PS1 control mice. GSEA is a method to identify classes of genes or proteins that over-represented in a large set of genes or proteins [90]. We conducted GSEA analysis to assess the enrichment of annotated gene sets in KEGG, Reactome and STRING local network clusters. KEGG pathway enrichment analysis was conducted using DAVID (http://david.abcc.ncfcrf.gov/) [91], which is an online tool providing a comprehensive set of functional annotations to understand biological meaning behind large list of genes. Reactome GSEA analysis was conducted using ReactomeFIViz (https://reactome.org/tools/reactome-fiviz) [92], which is a cytoscape app for pathway and network-based data analysis. STRING local network cluster enrichment analysis was conducted using STRING (http://string-db.org). KEGG is a database regarding genomes, biological pathways, diseases, drugs and chemical substances (http://www.genome/ad.jp/kegg) [93]. Reactome (http://www.reactome.org) is an open source, expert-authored, peer-reviewed, manually curated database of reactions, pathways and biological processes. STRING (http://string-db.org) database provides a critical assessment and integration of protein–protein interaction (PPI), including direct (physical) and indirect (functional) associations in a given organism [94].

### Immunohistochemistry

After transcardial perfusion, mouse brains were immediately harvested and divided into two hemispheres. The left was immediately frozen on dry ice for biochemical analysis and right was immersed in freshly depolymerized 4% paraformaldehyde in PBS for 48 h at 4 °C and cryoprotected by immersion in 15% then 30% sucrose for 24 h at 4 °C. Fixed brains were sectioned coronally with a Cryostat (ThermoFisher) with 30-μm-thick sections serially collected and stored at −80 °C. Immunohistochemistry was performed using antibodies against pan-Aβ (1:500, rabbit polyclonal, Catalog no. 715800, Thermo Fisher Scientific), Iba1 (1:1000, rabbit polyclonal, Catalog no. 01919741, Wako) and doublecortin (Dcx) (1:500, goat polyclonal, Catalog no. Sc8066, Santa Cruz Biotechnology). For immunodetection, biotin-conjugated anti-rabbit or anti-goat IgG secondary antibody was used followed by a tertiary incubation with Vectastain ABC Elite kit (Catalog no. PK6100, Vector Laboratories). 1% Thioflavin-S in 50% ethanol was used for counterstaining of compact amyloid plaque (Catalog no. T1892, Sigma). For each of the immunohistochemical staining, six sections/slide were collected at eight intervals and were used for each of the experimental groups. Slides were masked and coded and Aβ occupied area was calculated using Cavalieri estimator probe (grid spacing 15 μm), while the number of Iba1-reactive microglia and Dcx-positive (Dcx+) neuroprogenitor cells were counted using the optical fractionator module of Stereo Investigator system (MBF Bioscience) as described earlier [9]. Briefly, a high-sensitivity digital camera (OrcaFlash2.8, Hamamatsu C11440-10C, Hamamatsu, Japan) interfaced with a Nikon Eclipse 90i microscope (Nikon, Melville, NY, USA) was used. Within the Stereo Investigator program, the contour in each section was delineated using a tracing function. While sections showed tissue shrinkage along the anteroposterior axis, the extent of shrinkage between different animals was considered similar. The dimensions for the counting frame (120 × 100 μm) and the grid size (245 × 240 μm) were set. The z-plane focus was adjusted at each section for clarity. Cells were quantified by the fractionator with marked positive cells in each counting frame. Based on the set parameters and marked cell counts, Stereo Investigator computed cell population estimates for comparison between groups.

### Western blot analysis

Brain cortical tissues were homogenized using lysis buffer containing 50 mM Tris–HCl (pH 8.0), 150 mM NaCl, 50 mM EDTA, 1% Triton X-100, and a mixture of protease/phosphatase inhibitor (Catalog no. 89901, Thermo Fisher Scientific). The lysate was centrifuged at 20,000×*g* for 20 min at 4 °C, the supernatant collected and total protein quantified using micro-BCA kit (Catalog no. 23235, Thermo Fisher Scientific). For Western blots, 80 μg or 20 μg of tissue protein was incubated with β-mercaptoethanol containing Laemmli buffer at 100 °C for 5 min, followed by electrophoresis on SDS-polyacrylamide gel and transferred to polyvinylidene fluoride membrane (Immobilon-P, Catalog no. IPVH00010, Millipore). The membranes were blocked in 5% skim milk/TBST and incubated with primary antibodies to 22C11 (1:2000, Catalog no. MAB348, Millipore Sigma), 6E10 (1:2000, Catalog no. 803001, BioLegend), arginase 1 (1:300, Catalog no. 93668S, Cell Signaling Technology), iNOS (1:300, Catalog no. 13120S, Cell Signaling Technology), synaptophysin (1:1000, Catalog no. MAB5258, Millipore), PSD95 (1:1000, Catalog no. ab18258, Abcam) and β-actin (1:2000, Catalog no. A3854, Sigma) at 4 °C overnight, followed by 60 min incubation in 5% skim milk/TBST with horseradish peroxidase-conjugated anti-rabbit, mouse, or goat secondary antibodies (1:2000, Santa Cruz Biotechnology). Immunoreactive bands were detected using SuperSignal West Pico or Femto Chemiluminescent substrate, and images were captured using an iBlot750 Imager (Thermo Fisher Scientific). Immunoblots were quantified using ImageJ software (NIH) relative to β-actin expression.

### Aβ detection by ELISA

Mouse cortical tissues were homogenized in 50 mM Tris–HCl (pH 7.6) containing 150 mM NaCl and a protease/phosphatase inhibitor mixture. The homogenate was centrifuged at 20,000×*g* for 20 min at 4 °C and the supernatant was analyzed for human Aβ_1–40_ and Aβ_1–42_ by ELISA (Catalog nos. KHB3482 and KHB3442, Thermo Fisher Scientific) according to the manufacturer’s instructions.

### Statistical analysis

All data were normally distributed and presented as mean values ± standard errors of the mean (SEM). Comparisons of means between groups were analyzed by one-way ANOVA or two-way repeated measures ANOVA followed by Newman–Keuls post hoc test using GraphPad Prizm software version 8.0 (GraphPad Software, San Diego, CA). A value of *p* ≤ 0.05 was regarded as a significant difference.

## Supplementary Information

**Additional file 1: Figure S1.** Development of monoclonal Aβ-Th1 and Aβ-Th17 cells. **Figure S2.** Modelling and explicit solvent molecular dynamics simulations. **Figure S3.** Adoptive transfer of Aβ-Teffs did not affect T cell frequency in APP/PS1 mice. **Figure S4.** Transcriptomic analysis of immune genes. **Figure S5.** Western blot images.

## Data Availability

The datasets supporting the conclusions of this article are included in the manuscript. The transcriptomic data and analysis files are openly available in figshare at https://figshare.com/s/8f53b7738153efa5feeb.

## Abbreviations

Aβ: Amyloid beta
ACK: Ammonium–chloride–potassium
AD: Alzheimer’s disease
APP: Amyloid precursor protein
APCs: Antigen presenting cells
AUC: Area under curve
BBB: Blood–brain barrier
cDNA: Complementary DNA
CEST: Chemical exchange saturation transfer
CFSE: Carboxyfluorescein succinimidyl ester
CNS: Central nervous system
Dcx: Doublecortin
2DG: 2-Deoxy glucose
2DG6P: 2DG-6-phosphate
FBS: Fetal bovine serum
^18^F-FDG: ^18^F-radiolabeled fluorodeoxyglucose
FMO: Fluorescence-minus-one
FOXP3: Forkhead box P3
GSEA: Gene set enrichment analysis
IFNγ: Interferon gamma
IL2: Interleukin-2
IL17: Interleukin 17
iNOS: Inducible nitric oxide synthase
KEGG: Kyoto Encyclopedia of Genes and Gnomes
MD: Molecular dynamics
MHCII: Major histocompatibility complex II
NF-κB: Nuclear factor-kappa B
PBS: Phosphate-buffered saline
PET: Positron emission tomography
pMHC: Peptide–MHCII
PPI: Protein–protein interaction
PS1: Presenilin 1
RAWM: Radial arm water maze
RMSD: Root-mean-square deviation
RMSF: Root-mean-square fluctuations
RORγ: RAR-related orphan receptor gamma
SEM: Standard errors of the mean
STRING: Search Tool for the Retrieval of Interacting Genes/Proteins
Tbet: T-box expressed in T cells
TCR: T cell receptor
Teff: Effector T cell
Th1: Type 1 T helper
Th2: Type 2 Th
Th17: Type 17 Th
TIR: Toll/IL1 receptor
TNFα: Tumor necrosis factor alpha
Treg: Regulatory T cell
Tresp: T responder cell
