# Supplementary material for: CD4+ effector T cells accelerate Alzheimer’s disease in mice
Source: J Neuroinflammation. 2021 Nov 19;18:272. doi: 10.1186/s12974-021-02308-7 (PMC8603581; doi:10.1186/s12974-021-02308-7)
Supplement: Supplementary file 1 — Additional file 1: Figure S1. Development of monoclonal Aβ-Th1 and Aβ-Th17 cells. Figure S2. Modelling and explicit solvent molecular dynamics simulations. Figure S3. Adoptive transfer of Aβ-Teffs did not affect T cell frequency in APP/PS1 mice. Figure S4. Transcriptomic analysis of immune genes. Figure S5. Western blot images. [file 12974_2021_2308_MOESM1_ESM.pdf]

Additional file 1

CD4+ effector T cells accelerate Alzheimer's disease in mice

Jatin Machhi*[1], Pravin Yeapuri[1], Yaman Lu[1], Emma Foster[2], Rupesh Chikhale[3], Jonathan Herskovitz[4], Krista L. Namminga[1], Katherine E. Olson[1], Mai Mohamed Abdelmoaty[5,6], Ju Gao[1], Rolen M. Quadros[1,7], Tomomi Kiyota[8], Liang Jingjing[1], Bhavesh D. Kevadiya[1], Xinglong Wang[1], Yutong Liu[1,9], Larisa Y Poluektova[1], Channabasavaiah B. Gurumurthy[1,7], R Lee Mosley[1], Howard E Gendelman[1,5]*

[1]Department of Pharmacology and Experimental Neuroscience, University of Nebraska Medical Center, Omaha, NE 68198, USA
[2]Department of Biological Sciences, Northern Kentucky University, Highland Heights, KY 41099, USA
[3]University College London School of Pharmacy, Bloomsbury, London WC1E 6DE, UK
[4]Department of Pathology and Microbiology, College of Medicine, University of Nebraska Medical Center, Omaha, NE 68198, USA
[5]Department of Pharmaceutical Sciences, College of Pharmacy, University of Nebraska Medical Center, NE 68198, USA
[6]Therapeutic Chemistry Department, Pharmaceutical and Drug Industries Research Division, National Research Centre, Giza, Egypt
[7]Mouse Genome Engineering Core Facility, Vice Chancellor for Research Office, University of Nebraska Medical Center, Omaha, Nebraska, USA
[8]Department of Safety Assessment, Genentech Inc., South San Francisco, CA 94080, USA
[9]Department of Radiology, University of Nebraska Medical Center, Omaha, NE 68198, USA

Corresponding authors:

Howard E. Gendelman, Department of Pharmacology and Experimental Neuroscience, University of Nebraska Medical Center, Omaha, NE 6898-5880; phone 402-559-8920; fax 402-559-3744.
Email hegendel@unmc.edu (for review and correspondence)

Jatin Machhi, Department of Pharmacology and Experimental Neuroscience, University of Nebraska Medical Center, Omaha, NE 6898-5880; phone 402-559-2779.
Email jatin.machhi@unmc.edu (shared correspondence).

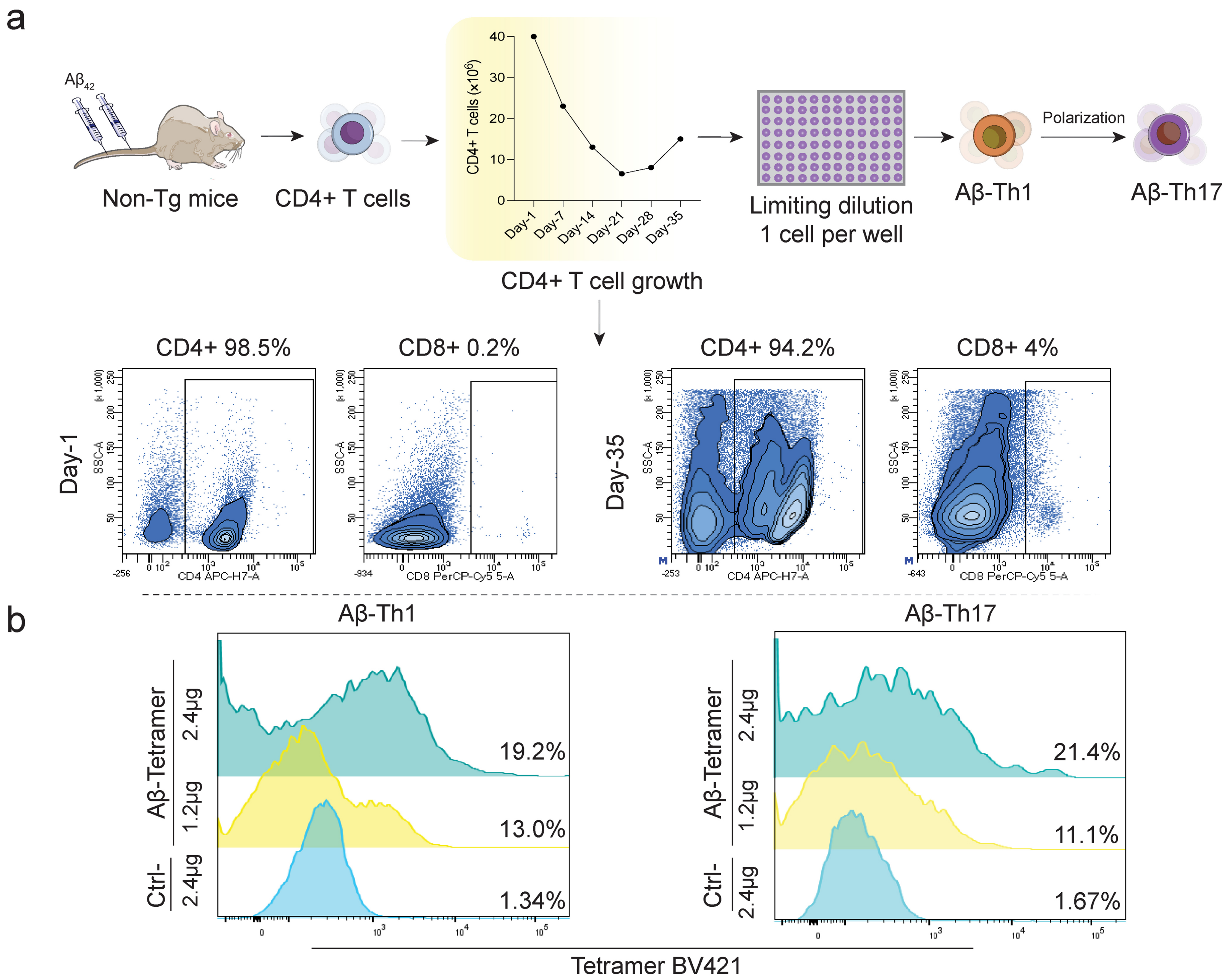
a
Aβ42
Non-Tg mice
CD4+ T cells
CD4+ T cells (×10^6)
Day-1
Day-7
Day-14
Day-21
Day-28
Day-35
CD4+ T cell growth
Limiting dilution
1 cell per well
Aβ-Th1
Polarization
Aβ-Th17
CD4+ 98.5%
CD8+ 0.2%
CD4+ 94.2%
CD8+ 4%
Day-1
Day-35
SSC-A
CD4 APC-H7-A
CD8 PerCP-Cy5 5-A
b
Aβ-Th1
Aβ-Th17
Aβ-Tetramer
2.4μg
1.2μg
Ctrl-
2.4μg
19.2%
13.0%
1.34%
21.4%
11.1%
1.67%
Tetramer BV421

Fig. S1. Development of monoclonal Aβ-Th1 and Aβ-Th17 cells. *(a) Workflow showing development of antigen-specific T cells. Non-Tg mice immunized with $A\beta_{1-42}$ followed by isolation of CD4+ T cells from spleen and lymph nodes. In vitro culture of CD4+ T cells in the presence of feeder cells and $A\beta_{1-42}$ for enrichment of antigen-rich population. Growth pattern of CD4+ T cells over in vitro incubation where cells growth declined for a several weeks followed by increased cell number without compromising CD4+ T cell purity (shown as % of CD3+ cells). Limiting dilution culture to seed as low as 1 cell per well in presence of feeder cells, $A\beta_{1-42}$, and IL2 to obtain Aβ-Th1 cell clone. Aβ-Th1 cells polarized into Aβ-Th17 cells using conditional culture media in presence of feeder cells and $A\beta_{1-42}$. (b) Staining of three-month propagated Aβ-Th1 and Aβ-Th17 cells with two concentrations of MHCII-IA^{b}-KLVFFAEDVGSNKGA (Aβ T cell epitope) tetramer after incubation at 37 °C for 3 hr. Control tetramer MHCII-IA^{b}-PVSKMRMATPLLMQA (Ctrl) was used to determine non-specific binding of T cells.*

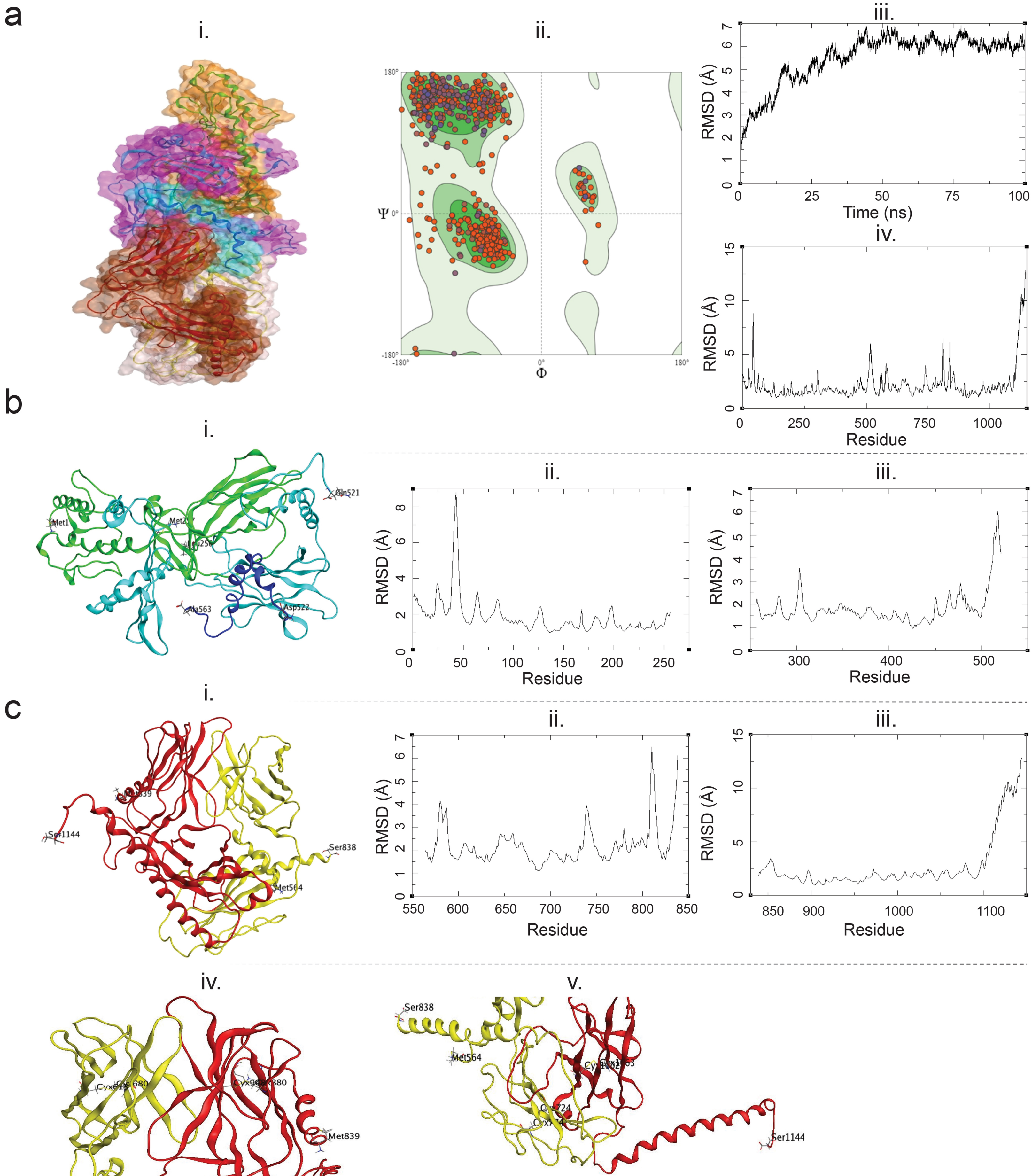

a
i.
ii.
iii.
iv.
Ψ
Φ
180°
-180°
0°
RMSD (Å)
Time (ns)
0
25
50
75
100
Residue
0
250
500
750
1000
b
Met1
Gln521
Leu256
Ala563
Asp522
RMSD (Å)
Residue
0
50
100
150
200
250
300
400
500
c
Ser1144
Ser838
Met564
RMSD (Å)
Residue
550
600
650
700
750
800
850
900
1000
1100
v.
Met839
Ser838
Met564
Ser1144

***Fig. S2. Modelling and explicit solvent molecular dynamics simulations**. (a) (i) TCR-pMHC complex with surface interactions (post MD production); (ii) Ramachandran plot for the optimized and energetically stabilized model; (iii) RMSD plot for the TCR-pMHC complex for 100 ns MD simulation; (iv) RMSF for complete TCR-pMHC complex over the period of 100 ns MD simulation. (b) (i) pMHC complex from the modelled complex; RMSF for the (ii) MHCα chain and (iii) MHCβ chain. (c) (i) TCR complex; (ii) RMSF of TCRα chain and (iii) TCRβ chain from the complex. (iv) Close-up of the Fab (fragment antigen binding) and (v) the Fc (fragment crystallizable) regions.*

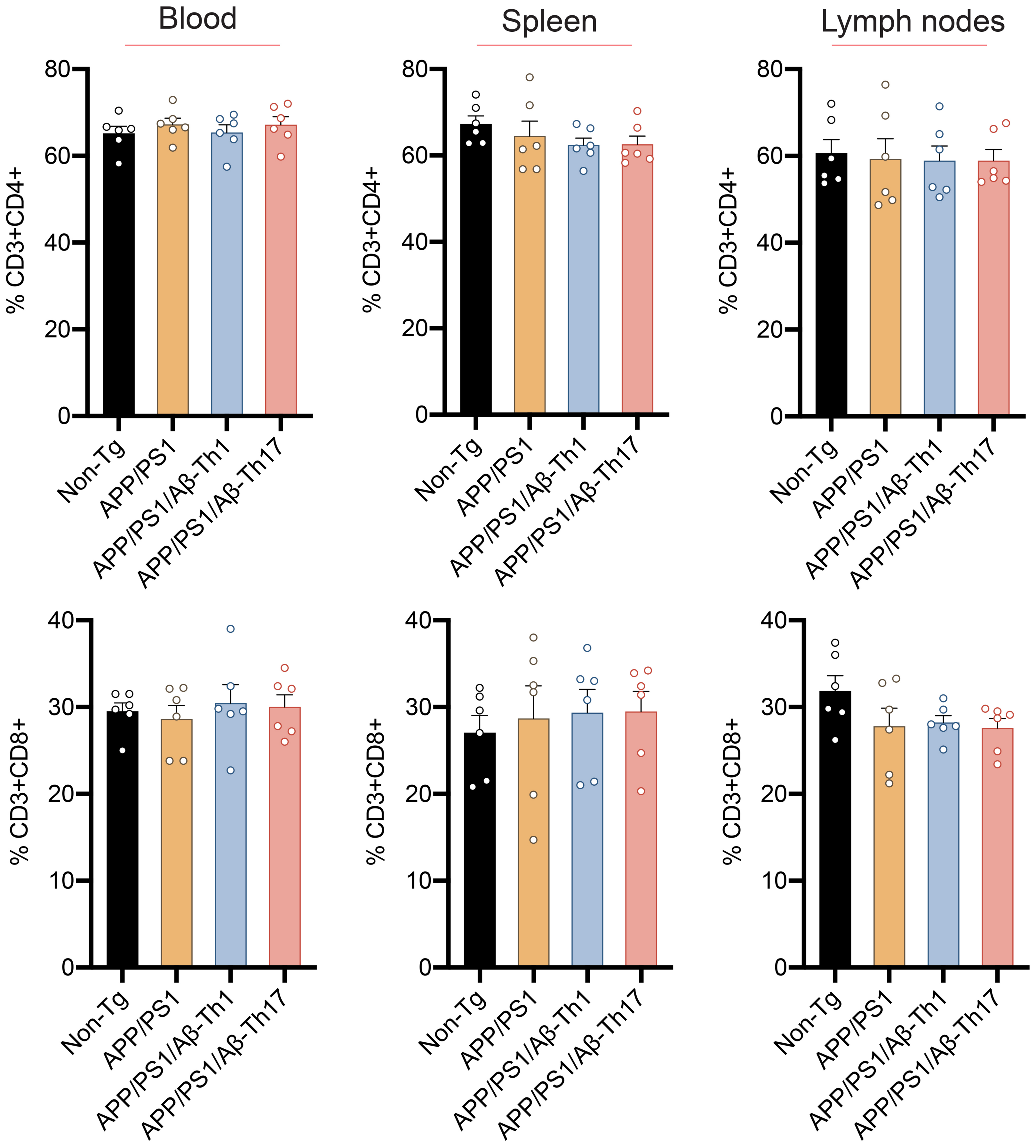

Blood
Spleen
Lymph nodes
% CD3+CD4+
% CD3+CD8+
0
20
40
60
80
0
10
20
30
40
Non-Tg
APP/PS1
APP/PS1/Aβ-Th1
APP/PS1/Aβ-Th17

Fig. S3. Adoptive transfer of Aβ-Teffs did not affect T cell frequency in APP/PS1 mice. *Frequency of CD3+CD4+ and CD3+CD8+ T cells in blood, spleen and lymph nodes by flow cytometric analysis for n=6 mice per group. One-way ANOVA followed by Newman–Keuls post-hoc test was used to determine statistical significance.*

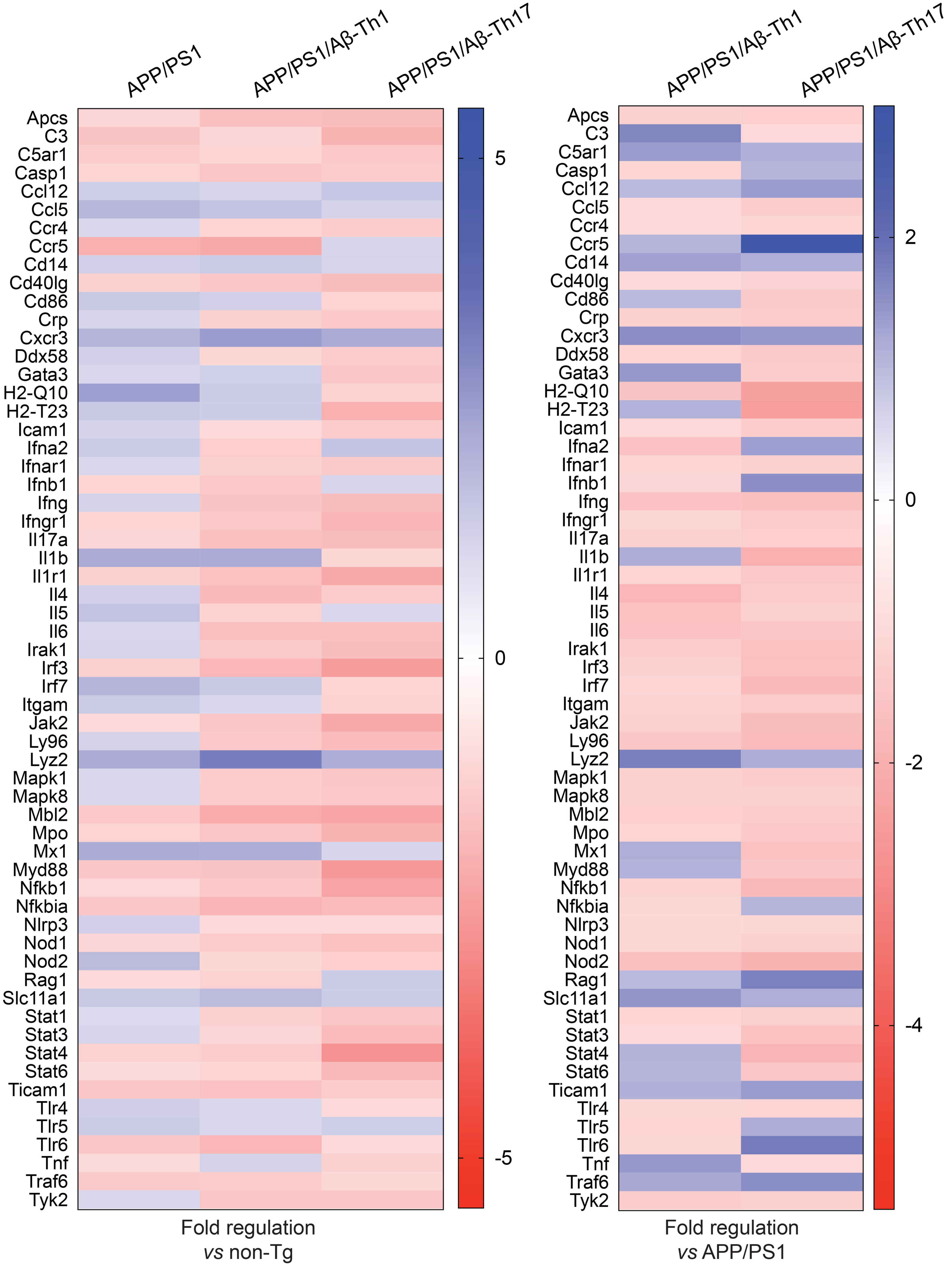

APP/PS1
APP/PS1/Aβ-Th1
APP/PS1/Aβ-Th17
Apcs
C3
C5ar1
Casp1
Ccl12
Ccl5
Ccr4
Ccr5
Cd14
Cd40lg
Cd86
Crp
Cxcr3
Ddx58
Gata3
H2-Q10
H2-T23
Icam1
Ifna2
Ifnar1
Ifnb1
Ifng
Ifngr1
Il17a
Il1b
Il1r1
Il4
Il5
Il6
Irak1
Irf3
Irf7
Itgam
Jak2
Ly96
Lyz2
Mapk1
Mapk8
Mbl2
Mpo
Mx1
Myd88
Nfkb1
Nfkbia
Nlrp3
Nod1
Nod2
Rag1
Slc11a1
Stat1
Stat3
Stat4
Stat6
Ticam1
Tlr4
Tlr5
Tlr6
Tnf
Traf6
Tyk2
5
0
-5
Fold regulation
vs non-Tg
APP/PS1/Aβ-Th1
APP/PS1/Aβ-Th17
2
0
-2
-4
Fold regulation
vs APP/PS1

Fig. S4. Transcriptomic analysis of immune genes. *Gene expression of different innate and adaptive immune genes from the hippocampus of each treatment group were assessed in Qiagen RT*^{2}*-PCR arrays. Genes less affected and not covered in* ***Fig. 5c*** *are summarized here. (left) Gene expression in different APP/PS1 mice compared to non-Tg mice. (right) Gene expression in APP/PS1/Aβ-Th1 and APP/PS1/Aβ-Th17 mice compared to untreated APP/PS1 mice for n=4 mice per group. Fold changes in different innate and adaptive immune genes are presented as heat maps.*

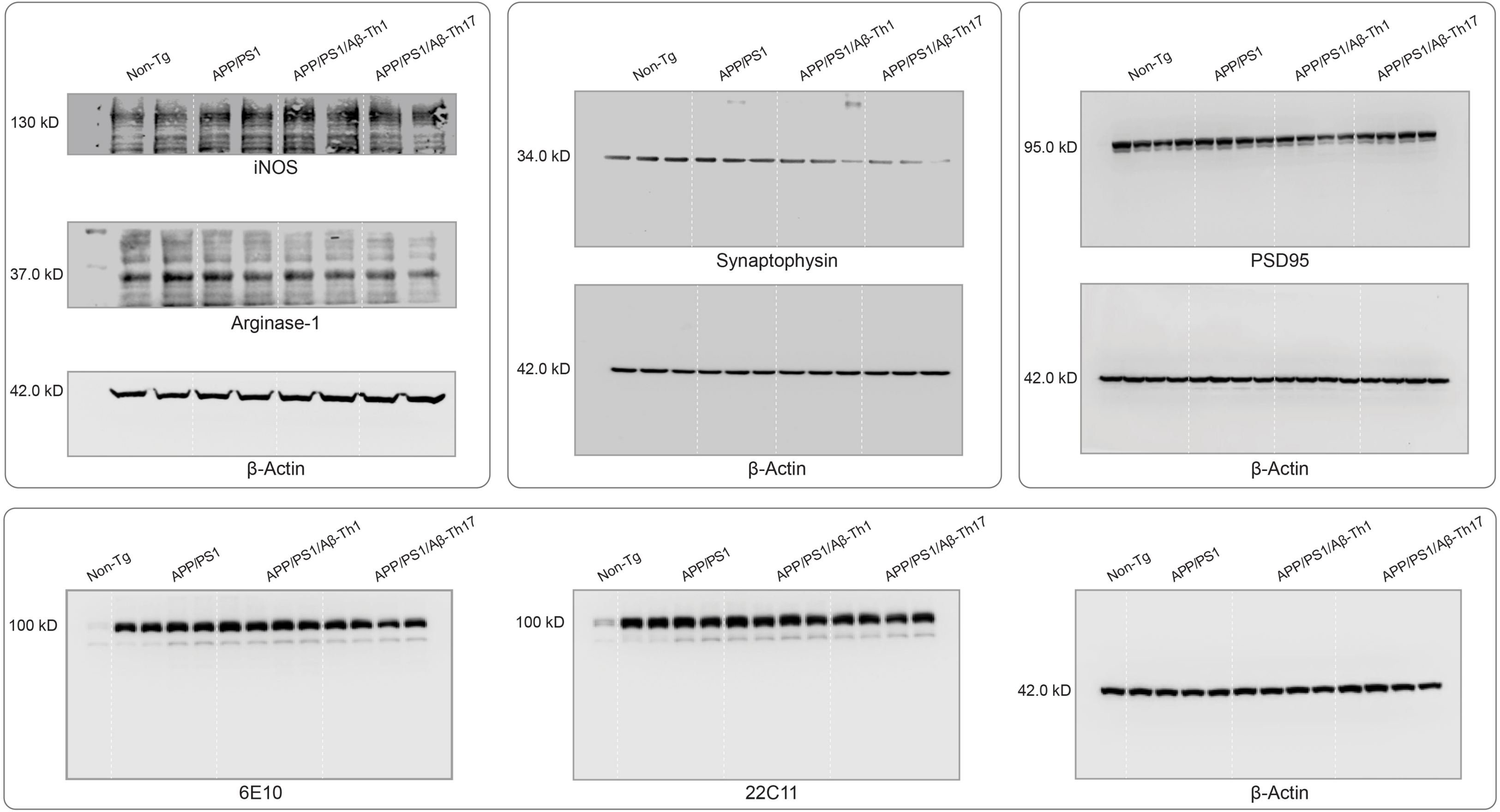
Non-Tg
APP/PS1
APP/PS1/Aβ-Th1
APP/PS1/Aβ-Th17
130 kD
iNOS
37.0 kD
Arginase-1
42.0 kD
β-Actin
34.0 kD
Synaptophysin
42.0 kD
β-Actin
95.0 kD
PSD95
42.0 kD
β-Actin
100 kD
6E10
100 kD
22C11
42.0 kD
β-Actin

Fig. S5. Western blot images. *Full blot image of iNOS, arginase-1, synaptophysin, PSD95, 6E10, 22C11 and β-actin protein expression determined from cortical tissues of each treatment group.*
